# Neutrophils Driving Unconventional T Cells Mediate Resistance against Murine Sarcomas and Selected Human Tumors

**DOI:** 10.1016/j.cell.2019.05.047

**Published:** 2019-07-11

**Authors:** Andrea Ponzetta, Roberta Carriero, Silvia Carnevale, Marialuisa Barbagallo, Martina Molgora, Chiara Perucchini, Elena Magrini, Francesca Gianni, Paolo Kunderfranco, Nadia Polentarutti, Fabio Pasqualini, Sabrina Di Marco, Domenico Supino, Clelia Peano, Ferdinando Cananzi, Piergiuseppe Colombo, Silvana Pilotti, Suliman Yousef Alomar, Eduardo Bonavita, Maria Rosaria Galdiero, Cecilia Garlanda, Alberto Mantovani, Sebastien Jaillon

**Affiliations:** 1 Department of Biomedical Sciences, Humanitas University, 20090 Pieve Emanuele, Italy; 2 Humanitas Clinical and Research Center, 20089 Rozzano, Italy; 3 Institute for Cancer Genetics, Columbia University, New York, NY 10032, USA; 4 Institute of Genetic and Biomedical Research, UoS Milan, National Research Council, 20089 Rozzano, Italy; 5 Surgical Oncology Unit, Humanitas Clinical and Research Center, Via Manzoni 56, 20089 Rozzano, Italy; 6 Pathology Department, Fondazione IRCCS Istituto Nazionale Tumori, 20133 Milan, Italy; 7 Zoology Department College of Science, King Saud University, 12372 Riyadh, Saudi Arabia; 8 Cancer Research UK Manchester Institute, The University of Manchester, Alderley Park SK10 4GT, UK; 9 Department of Translational Medical Sciences and Center for Basic and Clinical Immunology Research (CISI), University of Naples Federico II, 80138 Naples, Italy; 10 The William Harvey Research Institute, Queen Mary University of London, London EC1M 6BQ, UK

**Keywords:** neutrophils, tumor immunology, unconventional T cells, soft tissue sarcomas, innate immunity, carcinogenesis, interleukin-12, macrophages

## Abstract

Neutrophils are a component of the tumor microenvironment and have been predominantly associated with cancer progression. Using a genetic approach complemented by adoptive transfer, we found that neutrophils are essential for resistance against primary 3-methylcholantrene-induced carcinogenesis. Neutrophils were essential for the activation of an interferon-γ-dependent pathway of immune resistance, associated with polarization of a subset of CD4^−^ CD8^−^ unconventional αβ T cells (UTC_αβ_). Bulk and single-cell RNA sequencing (scRNA-seq) analyses unveiled the innate-like features and diversity of UTC_αβ_ associated with neutrophil-dependent anti-sarcoma immunity. In selected human tumors, including undifferentiated pleomorphic sarcoma, *CSF3R* expression, a neutrophil signature and neutrophil infiltration were associated with a type 1 immune response and better clinical outcome. Thus, neutrophils driving UTC_αβ_ polarization and type 1 immunity are essential for resistance against murine sarcomas and selected human tumors.

## Highlights

• Neutrophils mediate antitumor response by sustaining an IL-12/IFNγ-dependent pathway
• Neutrophils are essential for unconventional αβ T cell (UTCαβ) type 1 polarization
• Type 1 UTC_αβ_ possess an innate-like phenotype and display antitumor potential *in vivo*
• Neutrophil infiltration is associated with good prognosis in selected human tumors

## Graphical Abstract

**Figure:**
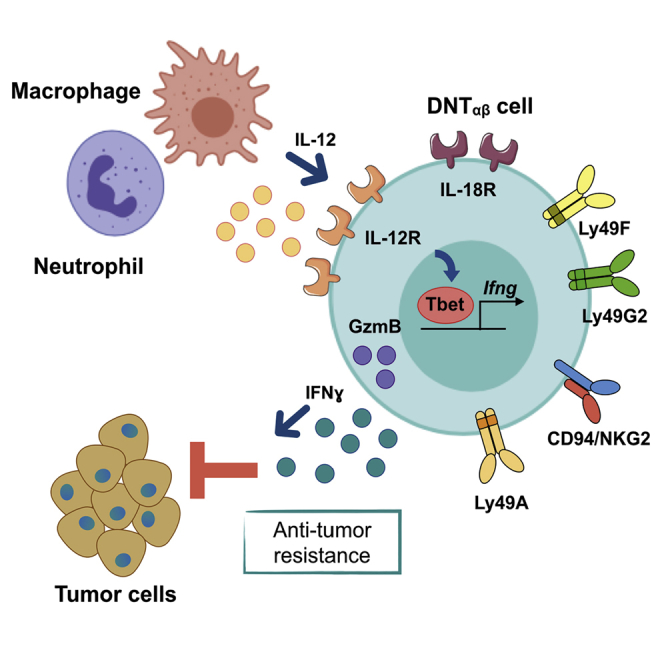

## Teaser

Tumor-associated neutrophils (TANs) have mainly been portrayed as tumor-promoters. Here, we describe a novel antitumor pathway in which TANs promote IL-12 production by macrophages, leading to type 1 polarization of a subset of unconventional αβ T cell (UTC_αβ_). Type 1 UTC_αβ_ possess an innate-like phenotype and antitumor potential *in vivo*. In selected human tumors, neutrophil infiltration is associated with type 1 immunity and better clinical outcome.

## Introduction

Neutrophils are the most abundant cell type in human peripheral blood and represent the first line of defense against invading microorganisms (Kolaczkowska and Kubes, 2013). Neutrophils play an important role in the activation and orchestration of acute inflammatory reactions (Borregaard, 2010, Ley et al., 2018). Moreover, neutrophils have emerged as important players in the regulation of innate and adaptive immunity and in chronic inflammation (Mantovani et al., 2011, Nicolás-Ávila et al., 2017).

Neutrophils are present in the tumor microenvironment (TME) and their function is regulated by signals produced by cancer cells and immune cells (Coffelt et al., 2016, Eruslanov et al., 2017, Ponzetta et al., 2017). Neutrophils and the myeloid growth factor granulocyte-colony stimulating factor (G-CSF) have predominantly been associated with tumor progression (Coffelt et al., 2016, Wculek and Malanchi, 2015). On the other hand, unleashed neutrophilic effectors have also been reported to mediate anti-cancer resistance (Colombo et al., 1991, Finisguerra et al., 2015, Fridlender et al., 2009, Granot et al., 2011, Massara et al., 2018, Sagiv et al., 2015, Singhal et al., 2016). For instance, neutrophils have been shown to regulate the function of conventional CD4^+^ and CD8^+^ tumor-infiltrating T cells with activating or suppressive effects, thus influencing tumor growth (Mantovani et al., 2011, Nicolás-Ávila et al., 2017). In addition to conventional T cells, neutrophils can also modulate the activation of γδ T cells, regulating their interleukin (IL)-17A production in cancer (Coffelt et al., 2015).

Available evidence on the role of neutrophils in carcinogenesis and tumor progression is essentially based on antibody-mediated cell depletion (Coffelt et al., 2015, Fridlender et al., 2009, Granot et al., 2011, Ponzetta et al., 2017). Given the intrinsic limitations of this approach including duration, specificity, and perturbation of the system (Faget et al., 2018, Granot et al., 2011, Moses et al., 2016), we set out to assess the role of neutrophils using a genetic strategy and a classic model of 3-methylcholanthrene (3-MCA)-induced carcinogenesis (Bonavita et al., 2015, Kaplan et al., 1998, Shankaran et al., 2001). We took advantage of genetic deficiency of G-CSF-R, a strategy analogous to that used to dissect the role of tumor-associated macrophages (TAMs) (Lin et al., 2001).

Unexpectedly, we found that neutrophils mediate resistance against primary carcinogenesis. Neutrophil-driven antitumor resistance was dependent on interferon-γ (IFNγ) produced by T cells. Neutrophil deficiency was associated with a selective impairment of type 1 polarization and IFNγ production by a subset of unconventional CD4^−^ CD8^−^ αβ T cells (UTC_αβ_). As assessed by flow cytometry and single-cell RNA sequencing (scRNA-seq) analyses, UTC_αβ_ were present in the sarcoma TME and were functionally regulated by neutrophils. Neutrophil infiltration was found to be associated with better prognosis and higher *IFNG* expression in human undifferentiated pleomorphic sarcomas (UPS) and in selected tumors. Thus, in murine sarcomas and selected human tumors, neutrophils are an essential component of type 1 antitumor immunity. More in general, the role of UTC_αβ_ in antitumor immunity may have been underestimated.

## Results

### Neutrophils Mediate Resistance against Primary 3-MCA Sarcomagenesis

**Figure S1:**
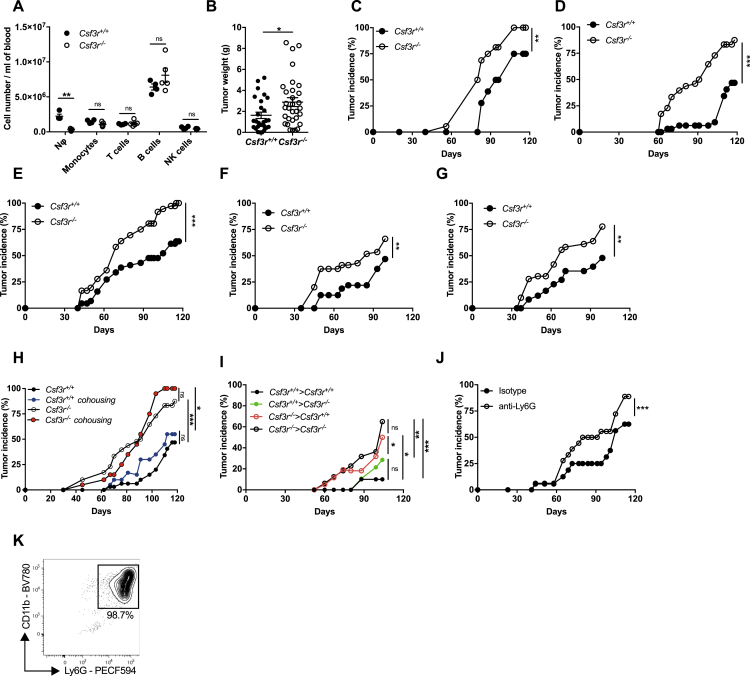
Neutrophil Deficiency in *Csf3r*^*−/−*^ Mice and Neutrophil Depletion in *Csf3r*^*+/+*^ Mice Increase Induction of 3-MCA-Sarcomagenesis, Related to Figure 1 (A) Absolute counts of main leukocyte populations in peripheral blood of healthy *Csf3r*^+/+^and *Csf3r*^−/−^mice. (B) *Csf3r*^+/+^and *Csf3r*^−/−^ sarcoma weight of mice sacrificed at the same time point (120 days after 3-MCA injection). (C-G) Tumor incidence of 5 representative experiments of 3-MCA induced sarcomas in *Csf3r*^+/+^and *Csf3r*^−/−^mice conducted over a period of 4 years. (H) Incidence of 3-MCA induced sarcomas in *Csf3r*^+/+^ and *Csf3r*^−/−^ mice bred separately or in cohousing conditions. (I) Incidence of 3-MCA induced sarcomas in bone marrow chimeras (donor > recipient). ( (J) Incidence of 3-MCA-induced sarcomas in *Csf3r*^+/+^ and *Csf3r*^−/−^ mice treated with anti-Ly6G antibody or with isotype control. (K) Representative dot plots showing the purity of naive neutrophils used in adoptive transfer experiments, gated on total isolated cells. (A-B) Data are mean ± SEM. ^∗^*p ≤* 0.05, ^∗∗^p ≤ 0.01 ^∗∗∗^p ≤ 0.001, ns, not statistically significant. (A) Two-tailed multiple Student’s t tests. (B) Two-tailed Mann-Whitney *U* test. (C-G), (J) Wilcoxon matched-pairs signed ranked test. (H-I) Friedman test with Dunn’s multiple comparison test. (A) n = 4 (*Csf3r*^+/+^) or n = 5 (*Csf3r*^−/−^) mice. (B) n = 27 (*Csf3r*^+/+^) or n = 31 (*Csf3r*^−/−^) mice. (C-G) n = 8-10 mice per group. (H) n = 8 (*Csf3r*^+/+^ separate), n = 12 (*Csf3r*^−/−^ separate), n = 5 (*Csf3r*^+/+^ cohoused), n = 8 (*Csf3r*^−/−^ cohoused) mice. (I) n = 11 *Csf3r*^+/+^ > *Csf3r*^−/−^, n = 12 *Csf3r*^−/−^ > *Csf3r*^−/−^, n = 13 *Csf3r*^−/−^ > *Csf3r*^+/+^, n = 14 *Csf3r*^+/+^ > *Csf3r*^+/+^. (J) n = 8 (*Csf3r*^+/+^ isotype) or n = 9 (*Csf3r*^−/−^ anti-Ly6G) mice. (A-J) One experiment performed. (B) Pooled data of four independent experiments. (C-G) 5 experiments out of twenty conducted over a period of 4 years.

**Figure 1:**
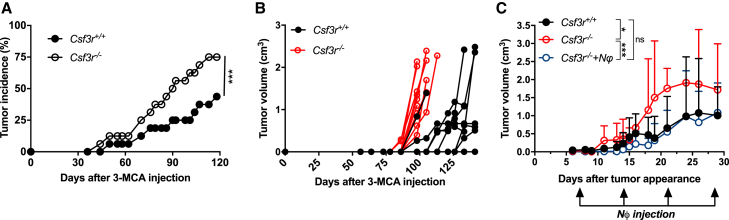
Neutrophils Mediate Resistance to 3-MCA-Induced Sarcomagenesis (A and B) Tumor (A) incidence and (B) growth of 3-MCA induced sarcomas in *Csf3r*^+/+^ and *Csf3r*^−/−^ mice. (C) Tumor growth of 3-MCA-induced sarcoma in *Csf3r*^+/+^ and *Csf3r*^−/−^ mice upon adoptive transfer of neutrophils (NΦ) in *Csf3r*^−/−^ mice. 3 × 10^6^ BM NΦ were intravenously (i.v.) transferred once a week (time points indicated by arrows) starting from the first day the tumor was palpable. Tumor growth is represented as volume over time after the first tumor observation. Data are mean ± SEM (A and B) or mean ± SD (C). ^∗^*p ≤* 0.05, ^∗∗∗^p ≤ 0.001. (A and C) Wilcoxon matched-pairs signed-rank test. See also Figures S1 and S2 and Table S1.

Genetic deficiency of the G-CSF-R (*Csf3r*^−/−^) caused a profound neutropenia in the peripheral blood of healthy mice (Figure S1A) (Liu et al., 1996). In the 3-MCA-induced sarcoma model, *Csf3r*^−/−^ mice showed earlier tumor development and increased tumor growth and weight, compared to wild type mice (Figures 1A, 1B, and S1B). The increased susceptibility of *Csf3r*^−/−^ to 3-MCA carcinogenesis was consistently observed in 20 experiments conducted over a period of 4 years, although as expected for primary carcinogenesis, with variability from experiment to experiment (6 experiments are shown in Figures 1A and S1C–S1G).

Dysbiosis is known to impact on carcinogenesis and anti-tumor responses (Zitvogel et al., 2015). However, cohousing did not affect sarcoma susceptibility (Figure S1H), excluding that a potential dysbiosis associated with *Csf3r* deficiency was involved in the observed phenotype.

In bone marrow chimeras, increased susceptibility to sarcomagenesis was associated with G-CSF-R deficiency in hematopoietic cells and neutrophil depletion by an anti-Ly6G antibody accelerated tumor development (Figures S1I and S1J). Adoptive transfer of bone marrow *Csf3r*^+/+^ neutrophils (purity >98.5%) (Figure S1K) into *Csf3r*^−/−^ sarcoma-bearing mice was sufficient to completely rescue tumor growth to the level of *Csf3r*^+/+^ controls (Figure 1C). Collectively, these results provide unequivocal genetic evidence that neutrophils mediate protection against primary 3-MCA sarcomagenesis.

### Tumor-Associated Neutrophils in *Csf3r*^**+/+**^ Mice Display an Activated Phenotype

**Figure S2:**
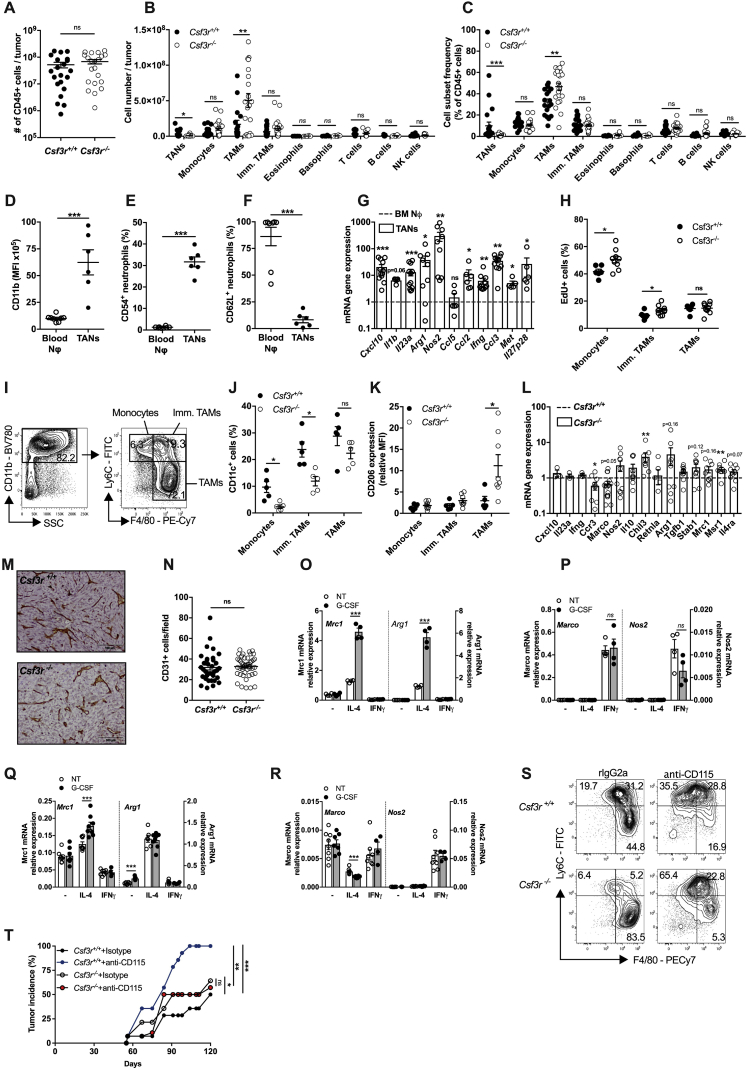
TANs in *Csf3r*^*+/+*^ Mice Display an Activated Phenotype; Role of Macrophages in the Increased Susceptibility of *Csf3r*^*−/−*^ to 3-MCA Sarcomagenesis, Related to Figures 1 and 2 (A-C) Number of sarcoma-infiltrating CD45^+^ cells (A), leukocyte cell subset frequencies (B) and absolute numbers (C) assessed by flow cytometry (tumor volume ≅ 2000 mm^3^). (D-F) Quantification by flow cytometry of CD11b, CD54 and CD62L expression in TANs and peripheral-blood neutrophils from *Csf3r*^+/+^ sarcoma-bearing mice. (G) mRNA gene expression in purified TANs. Gene expression was relative to Gapdh expression and normalized on the mean expression in naive bone marrow neutrophils (BM NΦ). (H) Proliferative activity of tumor-infiltrating myeloid subsets, assessed by flow cytometry (intracellular EdU staining). (I) Gating strategy for tumor-associated non-granulocytic myeloid populations. Cells represented in left dot plot are pregated on Aqua^-^/CD45^+^/Ly6G^-^ cells. (J-K) Flow cytometry analysis of CD206 and CD11c expression on tumor-associated non-granulocytic myeloid cells (L) mRNA expression of M1- and M2-selected genes in tumor-infiltrating TAMs. Gene expression was relative to Gapdh expression and normalized on the mean of expression found in *Csf3r*^+/+^ TAMs. (M-N) Immunohistochemical analysis and relative quantification of CD31^+^ cells in *Csf3r*^+/+^ and *Csf3r*^−/−^ sarcomas. 5 random fields per sample were counted. Scale bar, 100 μm. (O-R, mRNA expression of M1- and M2-related genes in BMDMs generated with M-CSF (O-P) or GM-CSF (Q-R) (see Methods). Gene expression was normalized on Gapdh expression. (S) Representative dot plots showing depletion of TAMs after treatment with anti-CD115 antibody. (T) Incidence of 3-MCA-induced sarcomas in *Csf3r*^+/+^ and *Csf3r*^−/−^ mice treated with anti-CD115 antibody or with isotype control. (A-F), (H), (J-K), (N-R). Data are mean ± SEM. ^∗^*p ≤* 0.05, ^∗∗^p ≤ 0.01 ^∗∗∗^p ≤ 0.001, ns, not statistically significant. (A), (D-F). Two-tailed Mann-Whitney *U* test. (B-C), (H), (J-K), (O-R) Two-tailed multiple Student’s t tests. (G), (L) Wilcoxon signed rank test. (T) Friedman test with Dunn’s multiple comparison test. (A) n = 21 mice per group. (B) n = 6 *Csf3r*^*+/+*^ eosinophils, basophils, n = 8 *Csf3r*^*−/−*^ eosinophils, basophils, n = 14 *Csf3r*^*+/+*^ T, NK cells, n = 9 *Csf3r*^*−/−*^ T, NK cells, n = 9 *Csf3r*^*+/+*^ B cells, n = 6 *Csf3r*^*−/−*^ B cells, n = 17 *Csf3r*^*+/+*^ TANs, monocytes, TAMs, immature TAMs, n = 20 *Csf3r*^*−/−*^ TANs, monocytes, TAMs, immature TAMs. (C) n = 6 *Csf3r*^*+/+*^ eosinophils, basophils, n = 8 *Csf3r*^*−/−*^ eosinophils, basophils, n = 18 *Csf3r*^*+/+*^ T, B, NK cells and TAMs, n = 19 TANs, monocytes, n = 20 *Csf3r*^*−/−*^ B cells, n = 21 *Csf3r*^*−/−*^ NK, T cells, n = 23 *Csf3r*^*−/−*^ TANs, monocytes, immature TAMs, TAMs. (D-F) n = 6 TANs, n = 8 blood neutrophils. (G) n = 5 Il1b, Met, n = 6 *Il27p28*, *Ccl5*, *Ccl2*, n = 9 *Nos2, Arg, Tnfa*, n = 11 *Ifng, Ccl3*, n = 12 *Cxcl10*. (H) n = 6 (*Csf3r*^+/+^) or n = 9 (*Csf3r*^−/−^) mice. (J-K) n = 5 mice. (L) n = 3 (*Cxcl10, Il23a, Ifng*), n = 7 (*Retnla*), n = 8 (*Chil3*), n = 9 (*Arg1, Stab1, Mrc1, Msr1, Il4ra*), n = 10 (*Ccr3, Nos2, Il10, Tgfb1*). (M-N) n = 7 (*Csf3r*^+/+^) or n = 9 (*Csf3r*^−/−^) mice. (O-P) n = 4 mice per group. (Q-R), n = 8 (NT, IL-4, IL-4 + G-CSF, IFNγ), n = 7 (G-CSF), n = 4 (IFNγ+G-CSF). (H) n = 7 (*Csf3r*^+/+^ Isotype, *Csf3r*^−/−^ Isotype, *Csf3r*^*−/−*^ anti-CD115) or n = 8 (*Csf3r*^+/+^ anti-CD115) mice per group. (A-C) Pooled data of four experiments are shown. (D-H), (M-N). (S-T) One experiment performed. (J-L) Pooled data from two (J-K) or three (L) experiments are shown. (O-R) Pooled data from two experiments.

The number of CD45^+^ cells infiltrating the tumor was similar in *Csf3r*^−/−^ and *Csf3r*^+/+^ mice (Figure S2A). Tumor-associated neutrophils (TANs) were virtually absent in *Csf3r*^−/−^ tumors (Figures S2B and S2C). In *Csf3r*^+/+^ sarcoma-bearing mice, TANs displayed an activated phenotype, characterized by increased expression of CD11b and CD54 and decreased expression of CD62L, compared to peripheral blood neutrophils (Figures S2D–S2F). mRNA expression of pro-inflammatory genes such as *Cxcl10*, *Il23a*, *Arg1*, *Nos2*, *Ccl2*, *Ifng*, *Ccl3*, *Met*, and *Il27p28* in sorted TANs was increased, compared to naive bone marrow neutrophils (Figure S2G). Thus, TANs presented a mixed phenotype expressing both N1-like (e.g., CD54, *Ccl3*, *Nos2*, and *Met*) and N2-like (e.g., *Arg1* and *Ccl2*) markers (Finisguerra et al., 2015, Fridlender et al., 2009).

### Neutrophil Deficiency Is Associated with Altered Polarization of Tumor-Associated Macrophages

The number of TAMs was significantly increased in *Csf3r*^−/−^ mice (Figures S2B and S2C). The increased frequency of TAMs in *Csf3r*^−/−^ mice was associated with increased proliferation observed in monocytes and immature macrophages (Figure S2H). In G-CSF-R-incompetent mice, monocytes and TAMs showed increased expression of the M2-associated marker CD206 and decreased expression of the M1-associated marker CD11c (Figures S2I–S2K), likely a reflection of defective type 1 immunity (see below). Gene expression analysis on sorted TAMs confirmed the increased expression of M2-like-related genes (Murray et al., 2014), including *Chil3*, *Tgfb1*, and *Msr1*, in *Csf3r*^−/−^-derived TAMs, while the expression of M1-related genes was either not modulated or decreased (Figure S2L). Intratumor vessel density was not altered in *Csf3r*^−/−^ mice (Figures S2M and S2N).

The G-CSF-R is expressed by monocytic lineage cells, although to a much lower extent compared to neutrophils (Christopher et al., 2011). However, G-CSF did not affect macrophage polarization by classical M1 or M2 signals (IFNγ and IL-4), and if anything, it skewed these cells in an M2-like direction (Figures S2O–S2R).

Therefore, the M2-like phenotype found in *Csf3r*^−/−^ TAMs is due to the absence of neutrophil-dependent response and not to lack of G-CSF-R signaling in the monocytic lineage.

### Neutrophils and Macrophages Cooperate to Promote an IFNγ-Dependent Antitumor Response

**Figure 2:**
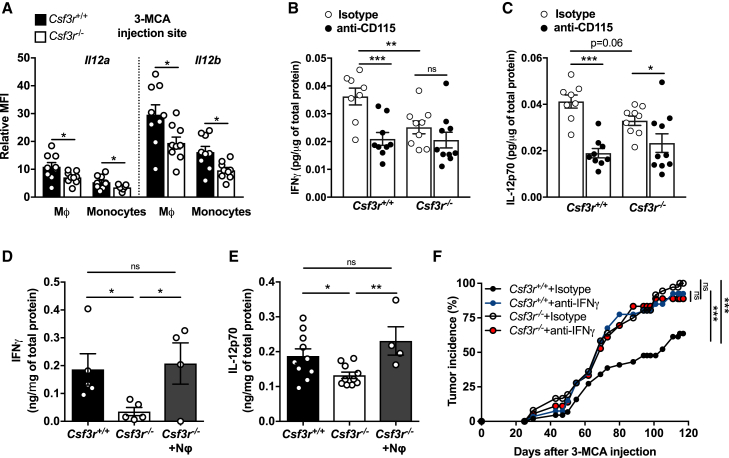
Neutrophils Mediate Tumor Resistance by Inducing a Macrophage-Dependent Activation of Type 1 Immunity (A) *Il12a* and *Il12b* mRNA expression (normalized on fluorescence minus one [FMO]) in myeloid cells infiltrating the 3-MCA injection site, analyzed by PrimeFlow RNA assay. (B and C) IFNγ (B) and IL-12p70 (C) concentrations at the 3-MCA injection site (10 days after 3-MCA administration) after treatment with anti-CD115 antibody or isotype control. (D and E) IFNγ (D) and IL-12p70 (E) concentrations in tumor homogenates after adoptive transfer of 3 × 10^6^ neutrophils once a week starting from the first day the tumor was palpable. (D) and (E) are two independent experiments conducted 12 months apart. (F) Incidence of 3-MCA induced sarcomas in *Csf3r*^+/+^ and *Csf3r*^−/−^ mice treated with anti-iFNγ antibody or with isotype control. (A–E) Data are mean ± SEM. ^∗^*p ≤* 0.05, ^∗∗^p ≤ 0.01, ^∗∗∗^p ≤ 0.001. (A) two-tailed multiple Student’s t tests. (B–E) One-way ANOVA. (F) Friedman test with Dunn’s multiple comparison test. See also Figure S2 and Table S1.

Macrophages obtained during 3-MCA carcinogenesis in G-CSF-R-competent mice expressed IL-12 and those from *Csf3r*^−/−^ mice displayed reduced *Il12a* and *Il12b* mRNA expression (Figure 2A). These results raised the issue of the role of TAMs in the increased susceptibility of *Csf3r*^−/−^ mice to 3-MCA carcinogenesis. As shown in Figures S2S and S2T, TAM depletion using an anti-CSF-1R (CD115) monoclonal antibody (mAb) did not rescue the phenotype of G-CSF-R-deficient mice, consistently with an essential role of neutrophils. Interestingly, TAM depletion increased carcinogenesis in G-CSF-R competent mice (Figure S2T) and drastically reduced the tissue levels of IL-12p70 and IFNγ (Figures 2B and 2C). As shown in Figures 2B–2E and Table S1, the increased sarcoma development observed in *Csf3r*^−/−^ neutrophil-deficient mice was associated with lower levels of IL-12p70 and IFNγ. Neutrophil adoptive transfer restored the expression of IL-12p70 and IFNγ in the TME of sarcoma-bearing *Csf3r*^−/−^ mice (Figures 2D and 2E). *In vivo* neutralization of IFNγ caused a dramatic increase of tumor incidence in *Csf3r*^+/+^ mice as previously reported (Kaplan et al., 1998, Koebel et al., 2007, Shankaran et al., 2001), but not in *Csf3r*^−/−^ mice (Figure 2F), abolishing the difference in sarcoma susceptibility between *Csf3r*^+/+^ and *Csf3r*^−/−^ control mice. These results indicate that the protective effect exerted by neutrophils was dependent on type 1 immunity and on the production of IFNγ.

### Neutrophils Are Essential for Type 1 Polarization of Unconventional αβ T Cells

**Figure S3:**
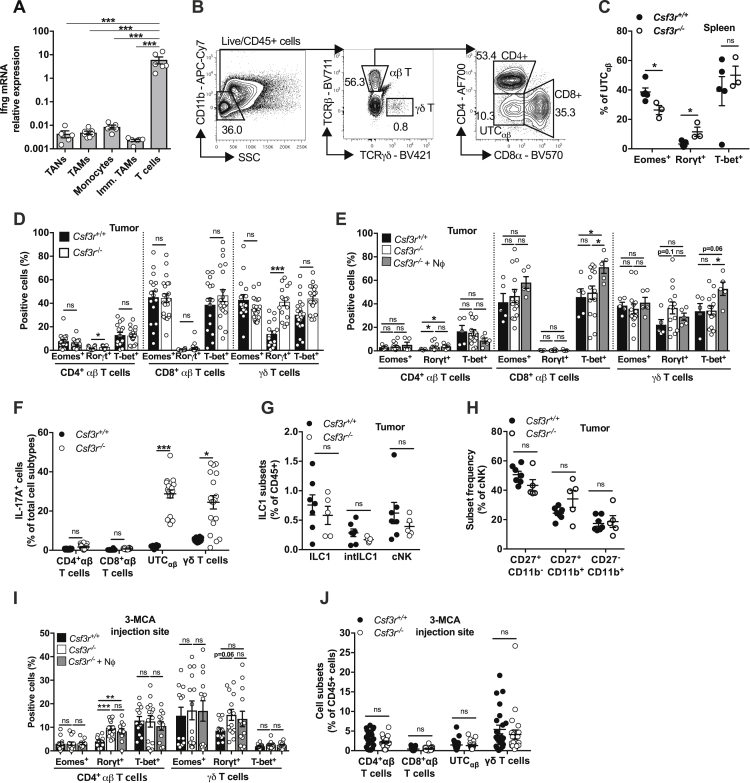
The Polarization of Conventional T Cells Is Not Altered in *Csf3r*^−/−^ Mice, Related to Figure 3 (A) *Ifng* mRNA expression determined by qPCR on sorted leukocyte subsets from *Csf3r*^+/+^ sarcomas. Gene expression was normalized on *Gapdh* expression. (B) Gating strategy used for tumor-infiltrating T cell subset characterization. Left panel represents Live/CD45^+^ cells. (C) Expression of Eomes, Rorγt and T-bet in splenic UTC_αβ_ from sarcoma-bearing *Csf3r*^+/+^ and *Csf3r*^−/−^ mice. (D) Expression of Eomes, Rorγt and T-bet in CD4+, CD8+ αβ T cells and γδ T cells infiltrating *Csf3r*^+/+^ and *Csf3r*^−/−^ sarcomas. (E) Expression of Eomes, Rorγt and T-bet in CD4+, CD8+ αβ T cells and γδ T cells infiltrating *Csf3r*^+/+^ and *Csf3r*^−/−^ sarcomas after neutrophil adoptive transfer. (F) Expression of IL-17A by tumor-infiltrating T cells stimulated *ex vivo* by PMA plus ionomycin. (G) Frequency of ILC1 subsets infiltrating *Csf3r*^+/+^ and *Csf3r*^−/−^ sarcomas determined by flow cytometry. (H) Maturation-related subset frequency within cNK cells infiltrating *Csf3r*^+/+^ and *Csf3r*^−/−^ sarcomas, determined by flow cytometry. (I) Expression of Eomes, Rorγt and T-bet in UTC_αβ_, CD4^+^ and γδ T cells infiltrating the 3-MCA injection site (10 days after administration of 3-MCA) after neutrophil adoptive transfer. CD8^+^ T cell polarization state could not be evaluated due to their low frequency in the tissue. (J) Frequency of T cell subsets infiltrating 3-MCA injection site (10 days after 3-MCA administration). (A), (C-J) Data are mean ± SEM. ^∗^*p ≤* 0.05, ^∗∗^p ≤ 0.01, ^∗∗∗^p ≤ 0.001; ns, not statistically significant. (A), (C), (F-H), (J) Two-tailed multiple Student’s t tests. (E, I) Kruskal-Wallis test with Dunn’s multiple comparison test. (D-E) 3x10^6^ neutrophils were i.v. transferred once a week starting from the first day the tumor was palpable. (I-J) 3x10^6^ neutrophils were i.v. transferred at days −1, 0, 1 and 9 with respect to 3-MCA administration. (A) n = 5 (TAN, Monocytes), n = 6 (Immature Monocytes, T cells), n = 7 (TAMs). (C) n = 5 (*Csf3r*^+/+^) or n = 3 (*Csf3r*^−/−^) mice. (D) n = 16 (*Csf3r*^+/+^) or n = 21 (*Csf3r*^−/−^) mice. (E) n = 5 (*Csf3r*^*+/+*^), n = 14 (*Csf3r*^−/−^), n = 5 (*Csf3r*^−/−^ + neutrophils) mice. (F) n = 10 (*Csf3r*^+/+^) or n = 18 (*Csf3r*^−/−^) mice. (G-H) n = 7 (*Csf3r*^+/+^) or n = 5 (*Csf3r*^−/−^) mice; i, n = 12 (*Csf3r*^*+/+*^), n = 14 (*Csf3r*^−/−^), n = 12 (*Csf3r*^−/−^ + Nϕ) mice. (J) n = 23 mice per group. (A), (C), (E), (G-H) One experiment performed. Pooled data from two (D, I) or three (F, J) experiments are shown.

**Figure 3:**
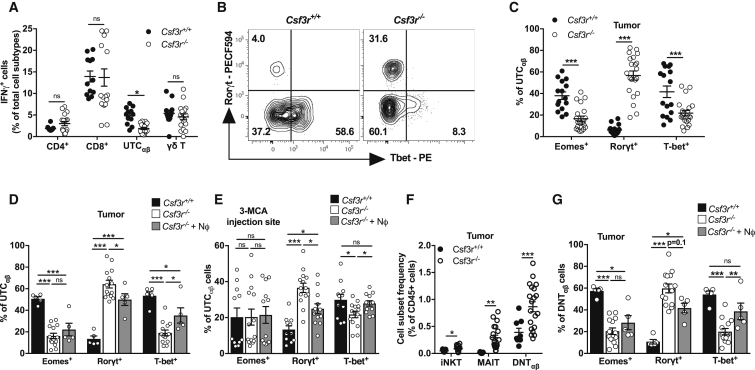
Neutrophils Drive DNT_αβ_ Type 1 Polarization (A) Expression of IFNγ by tumor-infiltrating T cells stimulated *ex vivo* by PMA plus ionomycin. (B) Representative dot plot showing Rorγt, and T-bet expression in UTC_αβ_ from *Csf3r*^+/+^ and *Csf3r*^−/−^ tumors. (C and D) Quantification of Eomes, Rorγt, and T-bet expression in UTC_αβ_ from *Csf3r*^+/+^ and *Csf3r*^−/−^ tumors (C) and in *Csf3r*^−/−^ mice after adoptive transfer of neutrophils (D). (E) Expression of Eomes, Rorγt, and T-bet in UTC_αβ_ infiltrating the 3-MCA injection site (10 days after administration of 3-MCA). (F) Quantification of iNKT, MAIT, and DNT_αβ_ frequencies among sarcoma-infiltrating CD45^+^ cells. (G) Polarization of tumor-associated DNT_αβ_ cells after neutrophil adoptive transfer. (D and G) 3 × 10^6^ neutrophils were transferred i.v. once a week starting from the first day the tumor was palpable. (E) 3 × 10^6^ neutrophils were transferred i.v. at days −1, 0, 1, and 9 with respect to 3-MCA administration. (A and C–G) Data are mean ± SEM. ^∗^p ≤ 0.05, ^∗∗^p ≤ 0.01, ^∗∗∗^p ≤ 0.001. (A, C, and F) Two-tailed multiple Student’s t tests. (D) One-way ANOVA. (E and G) Kruskal-Wallis test with Dunn’s multiple comparison test. See also Figures S3 and S4.

Having established that IFNγ played a key role in neutrophil-mediated resistance to 3-MCA carcinogenesis, it was important to identify the cellular source of this cytokine. Analysis of *Ifng* mRNA indicated T cells as the major source of this cytokine in the TME (Figure S3A). No difference was observed in IFNγ production in CD4^+^, CD8^+^, and γδ T cells between *Csf3r*^+/+^ and *Csf3r*^−/−^ mice (Figure 3A). In contrast, the frequency of IFNγ^+^ CD4^−^ CD8^−^ unconventional αβ T cells (UTC_αβ_) was drastically reduced (by 61.3% ± 11.4%; mean ± SEM in 2 experiments) in *Csf3r*^−/−^ tumors (Figure 3A). We then assessed the polarization of tumor-infiltrating CD3^+^ T cell subsets by flow cytometry (gating strategy in Figure S3B). UTC_αβ_ from *Csf3r*^−/−^ tumors displayed reduced expression of T-bet and Eomes and increased expression of Rorγt, indicating a skewing toward a type 3 activation state (Figures 3B and 3C). A trend toward a skewed UTC_αβ_ polarization was also observed in the spleen of *Csf3r*^−/−^ tumor-bearing mice, although to a minor degree compared to sarcoma-infiltrating cells (Figure S3C). γδ T cells from *Csf3r*^−/−^ sarcomas showed increased Rorγt expression, while Eomes and T-bet expression was not altered in this cell type (Figure S3D). Minor or no differences were observed in conventional CD4^+^ T cells and CD8^+^ T cells (Figure S3D). Neutrophil adoptive transfer reversed to a significant extent the polarization defect observed in UTC_αβ_ from *Csf3r*^−/−^ tumors (Figure 3D). Increased T-bet expression was also observed in γδ T cells and CD8^+^ T cells (Figure S3E). IL-17A expression was significantly increased in *Csf3r*^−/−^ UTC_αβ_ and γδ T cells, in agreement with their increased expression of Rorγt (Figure S3F). Natural killer (NK) cells are known to be important IFNγ producers (Vivier et al., 2018) and have been shown to undergo a functional conversion toward ILC1 during sarcoma progression (Gao et al., 2017), but no difference was observed in abundance or maturation state of the NK cell compartment in *Csf3r*^−/−^ tumors (Figures S3G and S3H).

Previous reports indicated that innate-like γδ T lymphocytes, represented an early source of IFNγ during the 3-MCA-induced sarcomagenesis (Gao et al., 2003). Here, the impaired type 1 activation state of UTC_αβ_ was observed as early as 10 days after 3-MCA injection, while little or no differences were observed in the polarization of γδ and CD4^+^ T cells (Figures 3E and S3I). The frequency of UTC_αβ_, γδ T cells, and conventional CD4^+^ T cells was not appreciably altered at the 3-MCA injection site (Figure S3J). It should be noted that at early times (10 days after 3-MCA injection), CD8^+^ T cells were virtually absent (Figure S3J). Bone marrow neutrophil transfer induced a complete rescue of T-bet expression and partially reduced the expression of Rorγt in *Csf3r*^−/−^ UTC_αβ_ present at the 3-MCA injection site, as early as 10 days after 3-MCA administration, indicating that neutrophils are an essential component of UTC_αβ_ polarization early in 3-MCA-induced sarcomagenesis (Figure 3E). In contrast, the polarization of other T cell subsets was not affected by neutrophil transfer at early time points (Figure S3I).

**Figure S4:**
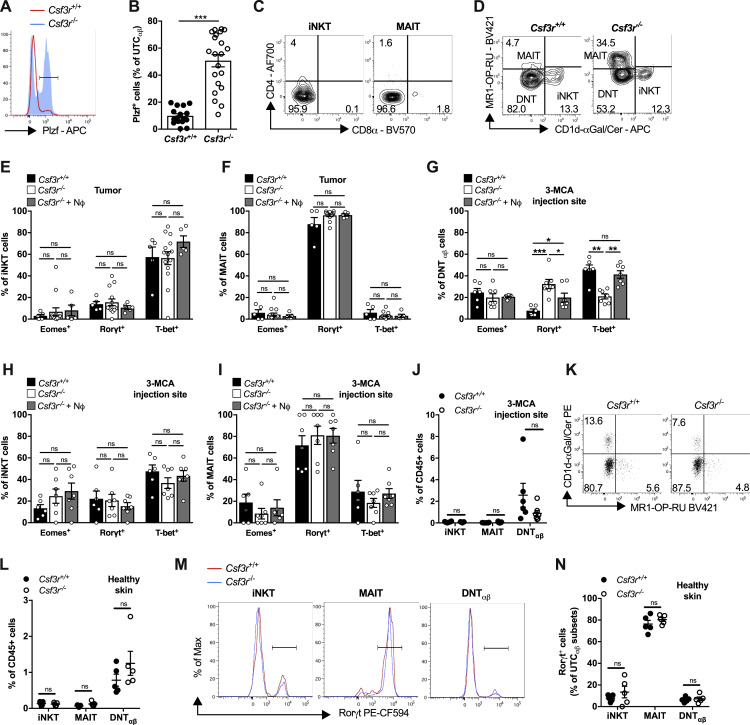
Frequency and Polarization of MAIT and iNKT Cells Are Not Altered in *Csf3r*^*−/−*^ Mice, Related to Figures 3 and 4 (A-B) Representative histogram and relative quantification of Plzf expression in tumor-infiltrating *Csf3r*^+/+^ and *Csf3r*^−/−^ UTC_αβ_, assessed by flow cytometry. (C) Representative dot plots from a *Csf3r*^−/−^ sarcoma. Expression of CD4 and CD8 on tumor-infiltrating iNKT and MAIT cells. (D) Representative plots showing iNKT, MAIT and DNT_αβ_ frequencies among sarcoma-infiltrating UTC_αβ_ in *Csf3r*^+/+^ and *Csf3r*^−/−^ mice. (E-F) Expression of Eomes, Rorγt and T-bet in sarcoma-infiltrating iNKT and MAIT cells after adoptive neutrophil transfer. (G-I) Expression of Eomes, Rorγt and T-bet in UTC_αβ_, iNKT and MAIT cells at the 3-MCA injection site (10 days after 3-MCA administration) after adoptive neutrophil transfer. (J) iNKT, MAIT and DNT_αβ_ frequencies among CD45^+^ cells infiltrating the 3-MCA injection site (10 days after administration of 3-MCA) in *Csf3r*^+/+^and *Csf3r*^−/−^ mice. (K-L) Representative dot plots (K) and quantification (L) of iNKT, MAIT and DNT_αβ_ frequencies among CD45+ cells in dorsal skin of healthy *Csf3r*^+/+^and *Csf3r*^−/−^ mice. (M-N) Representative histograms (M) and relative quantification (N) of Rorγt expression in iNKT, MAIT and DNT_αβ_ cells from dorsal skin of healthy *Csf3r*^+/+^and *Csf3r*^−/−^ mice. (B), (E-J), (L), (N) Data are mean ± SEM. ^∗∗∗^p ≤ 0.001; ^∗^p ≤ 0.05; ns, not statistically significant. (B), (J), (L), (N) Two-tailed multiple Student’s t tests. (E), (F), (I) Kruskal-Wallis test with Dunn’s multiple comparison test. (G-H) One-way ANOVA. (E-F) 3x10^6^ neutrophils were i.v. transferred once a week starting from the first day the tumor was palpable. (G-I) 3x10^6^ neutrophils were i.v. transferred at days −1, 0, 1 and 9 with respect to 3-MCA administration. (B) n = 16 (*Csf3r*^+/+^) or n = 21 (*Csf3r*^−/−^) mice. (E-F) n = 5 (*Csf3r*^+/+^), n = 14 (*Csf3r*^−/−^) or n = 5 (*Csf3r*^−/−^ + neutrophils). (G-I) n = 6 (*Csf3r*^+/+^), n = 7 (*Csf3r*^−/−^) or n = 7 (*Csf3r*^−/−^ + Nϕ) mice. (J) n = 6 (*Csf3r*^+/+^), n = 7 (*Csf3r*^−/−^) mice. (L), (N) n = 7 mice per group. (B) Two pooled experiments. (E-J), (L), (N) One experiment performed.

*Csf3r*^−/−^ UTC_αβ_ expressed high levels of Plzf (Figures S4A and S4B), a commonly expressed transcription factor in invariant natural killer T (iNKT) cells and mucosal-associated invariant T (MAIT) cells (Koay et al., 2016, Kovalovsky et al., 2008). These innate-like T cell subsets are also prevalently negative for CD4 and CD8 and can express Rorγt and T-bet (Cui et al., 2015, Engel et al., 2016, Rahimpour et al., 2015). Dissection of intratumor UTC_αβ_ into MAIT, iNKT, and a third cell subset, referred to as αβ double negative T (DNT_αβ_) cells, showed the heterogeneity of UTC_αβ_ in 3-MCA-treated mice (Figures S4C and S4D). We found increased frequencies of iNKT, MAIT, and DNT_αβ_ cells in *Csf3r*^−/−^ sarcomas, but only the polarization of DNT_αβ_ cells was altered in neutropenic mice (Figures 3F, 3G, S4E, and S4F). In the same line, neutrophil adoptive transfer modulated the polarization of DNT_αβ_ cells but not iNKT and MAIT cells both at the early time point (at the 3-MCA injection site, day 10) (Figures S4G–S4I) and in established tumors (Figures 3G, S4E, and S4F). On day 10, the frequency of UTC_αβ_ subsets was unaffected in *Csf3r*^−/−^ mice (Figure S4J). The presence and polarization of UTC_αβ_ subsets were not altered in the subcutaneous tissue of healthy mice (Figures S4K–S4N). Altogether, these data showed that neutrophils specifically regulated the polarization of DNT_αβ_ cells at early and late time points during carcinogenesis.

### Neutrophil-Dependent IL-12 Production Is Essential for IFNγ Expression in UTC_αβ_

**Figure 4:**
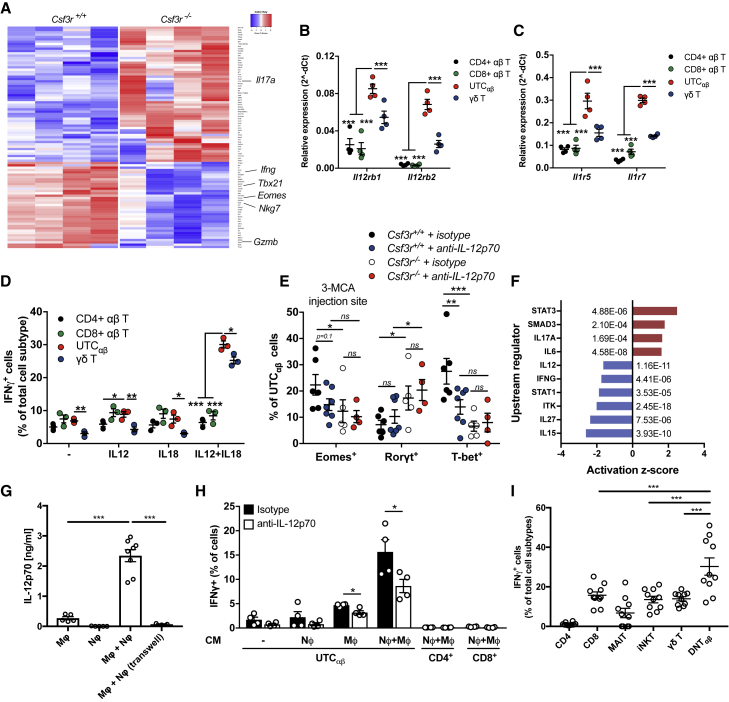
IL-12-Dependent Polarization and IFNγ Production Is Impaired in Tumor-Infiltrating UTC_αβ_ from *Csf3r*^−/−^ Mice (A) Heatmap showing differential transcriptional profiles of *Csf3r*^+/+^ and *Csf3r*^−/−^ tumor-associated UTC_αβ_. Differentially expressed genes (p ≤ 0.001) are shown (arrows indicate genes associated with effector functions). (B and C) mRNA expression of (B) *Il12rb1* and *Il12rb2* and (C) *Il1r5* and *Il1r7* in splenic T cell subsets isolated from untreated *Csf3r*^+/+^ mice. (D) Expression of IFNγ by splenic T cell subsets isolated from untreated *Csf3r*^+/+^ mice. (E) Eomes, Rorγt, and T-bet expression in UTC_αβ_ infiltrating the 3-MCA injection site after treatment with IL-12p70-neutralizing antibody or isotype control. (F) Predicted upstream regulators in tumor-infiltrating *Csf3r*^−/−^ UTC_αβ_ compared with tumor-infiltrating *Csf3r*^+/+^ UTC_αβ_, generated by IPA analysis. Associated p p value is shown for each regulator. (G) IL-12p70 levels detected by ELISA in supernatants of BMDM-neutrophil cocultures after stimulation with GM-CSF+CpG. (H) Expression of IFNγ in naive splenic αβ T cell subsets stimulated for 24 h with supernatants collected in (G) in the presence of IL-12p70-neutralizing antibody or isotype control, assessed by flow cytometry. (I) IFNγ production from *Csf3r*^+/+^ sarcoma-infiltrating T cells upon stimulation with IL-2+IL-12+IL-18 for 24 h assessed by flow cytometry. (B–I) Data are mean ± SEM. ^∗∗∗^p ≤ 0.001; ^∗∗^p ≤ 0.01; ^∗^p ≤ 0.05; ns, not statistically significant. (B–E and I) One-way ANOVA. (G and H) Two-tailed Student’s t test. See also Figures S4 and S5 and Tables S2 and S3.

**Figure S5:**
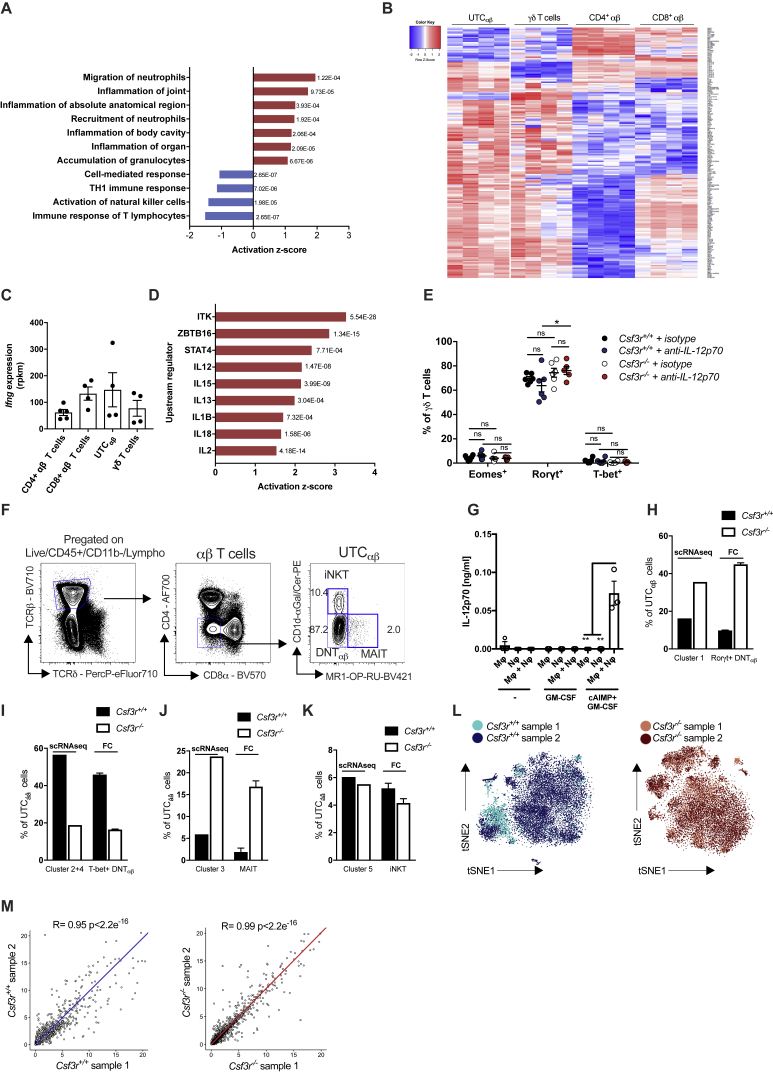
UTC_αβ_ from *Csf3r*^+/+^ Mice Show High Expression of Type 1 Immune Response-Related Genes and Are Responsive to IL-12, Related to Figures 4 and 5 (A) Diagram showing predicted upregulated and downregulated biological pathways using IPA software on *Csf3r*^−/−^ tumor-associated UTC_αβ_, compared to *Csf3r*^+/+^ tumor-associated UTC_αβ_. (red: upregulated pathways; blue: downregulated pathways). Associated *p-value* is shown for each pathway. (B) RNA-seq analysis of tumor-infiltrating T cell subsets isolated from *Csf3r*^+/+^ mice. Differentially expressed genes (p ≤ 0.001) in UTC_αβ_ compared with γδ T cells, CD4^+^ and CD8^+^ T cells are shown. Scale bar representing expression z-score in shown on the left. (C) Relative expression of *Ifng* mRNA in *Csf3r*^+/+^ T cell subsets determined by bulk RNA-seq. (D) Predicted upstream regulators in tumor-infiltrating *Csf3r*^+/+^ UTC_αβ_ compared with other tumor-infiltrating *Csf3r*^+/+^ T cell subsets, generated by IPA analysis; Associated *p-value* is shown for each regulator. (E) Expression of Eomes, Rorγt and T-bet in γδ T cells infiltrating the 3-MCA injection site after treatment with IL-12p70-neutralizing antibody or isotype control. (F) Representative gating strategy for identification of splenic *Csf3r*^+/+^ DNT_αβ_ cells. (G) IL-12p70 levels detected by ELISA in supernatants of BMDM-neutrophil cocultures after stimulation with GM-CSF+STING agonist cAIMP. (H-K) Frequency of indicated UTC_αβ_ subsets within total pool of tumor-infiltrating UTC_αβ_ cells in *Csf3r*^+/+^ and *Csf3r*^−/−^ mice analyzed by scRNaseq and by flow cytometry. (L) t-SNE projections showing overlap between *Csf3r*^+/+^ and *Csf3r*^−/−^ biological replicates. (M) Pearson correlation analyses of total gene expression averages between the 2 biological replicates across *Csf3r*^+/+^ and *Csf3r*^−/−^ conditions. (C, E, G–K) Data are mean ± SEM. ^∗∗∗^p ≤ 0.001; ^∗∗^p ≤ 0.01; ^∗^p ≤ 0.05; ns, not statistically significant. (E) One-way ANOVA, (G) two-tailed Student’s t test. (C) n = 4 per group. (E) n = 6 (*Csf3r*^+/+^ isotype), n = 6 (*Csf3r*^−/−^ isotype), n = 6 (*Csf3r*^+/+^ anti-iL-12p70), n = 5 (*Csf3r*^−/−^ anti-iL-12p70). (G) n = 3, (H-K) (flow cytometry data), n = 6 *Csf3r*^+/+^, n = 14 *Csf3r*^*−/−*^. (H-K) (scRNaseq data), pooled data from n = 2 *Csf3r*^+/+^ and n = 2 *Csf3r*^*−/−*^*mice*. (A-E, G-M) One experiment performed.

In an effort to better characterize the features of UTC_αβ_ associated with neutrophil-sustained anti-sarcoma type 1 immunity, bulk RNA-seq analysis of sorted sarcoma-infiltrating UTC_αβ_ was performed. 95 genes were differentially expressed between *Csf3r*^+/+^ and *Csf3r*^−/−^ UTC_αβ_ (Figure 4A; Table S2). The expression of *Il17a*, *Tbx21*, *Eomes*, and *Ifng* genes in *Csf3r*^−/−^ UTC_αβ_ was in line with data obtained by flow cytometry at the protein level with decreased expression of T-bet, Eomes, and IFNγ and increased expression of IL-17A (Figures 3A–3C and S3F). Ingenuity pathway analysis (IPA) highlighted the upregulation of pathways involved in inflammatory responses and neutrophil recruitment and downregulation of the pathway of Th1 immune response in *Csf3r*^−/−^ UTC_αβ_ (Figure S5A; Table S3). To identify the signaling pathways specifically activated on cancer-associated UTC_αβ_, we compared the transcriptome of tumor-associated *Csf3r*^+/+^ UTC_αβ_ with γδ T cells and conventional CD4^+^ and CD8^+^ T cells and found 190 differentially expressed genes (Figure S5B; Table S2). The *Ifng* mRNA levels detected in UTC_αβ_ isolated from *Csf3r*^+/+^ sarcomas were comparable to that expressed by CD8^+^ and γδ T cells (Figure S5C), indicating the significance of UTC_αβ_-derived IFNγ in the TME.

Upstream regulator analysis predicted the increased activation of STAT4, IL-18, and IL-12 pathways in tumor-associated UTC_αβ_, compared to other T cell subsets (Figure S5D; Table S3). qPCR performed on splenic T cell subsets from untreated mice confirmed the higher expression of *Il12rb1*, *Il12rb2*, *Il1r5*, and *Il1r7* in UTC_αβ_ compared to conventional T cells (Figures 4B and 4C). Accordingly, UTC_αβ_ produced higher IFNγ levels in response to IL-12 plus IL-18 stimulation, compared to other T cell subsets (Figure 4D). *In vivo* IL-12p70 neutralization skewed the polarization of *Csf3r*^+/+^ UTC_αβ_ to a T-bet^low^ phenotype (Figure 4E), while no effect was observed in γδ T cells (Figure S5E). Consistently, signaling related to several type 1 cytokines in *Csf3r*^−/−^ UTC_αβ_, including IL-12, were predicted to be significantly inhibited compared to *Csf3r*^+/+^ UTC_αβ_ (Figure 4F; Table S3).

The results discussed above suggest that in 3-MCA carcinogenesis neutrophils, in concert with macrophages, trigger a protective type 1 response involving IFNγ-producing UTC_αβ_ cells. In an effort to explore the cellular basis for this tripartite interaction, an *in vitro* coculture system was set up. For these studies, spleen-isolated UTC_αβ,_ which are composed by more than 85% of DNT_αβ_ cells, were used (Figure S5F). In an *in vitro* coculture model, neutrophils dramatically amplified IL-12 production by macrophages in response to triggering by cytokines and TLR9 agonist (Figure 4G) or, to a lesser extent, STING agonist (Figure S5G), which mimic conditions of tissue damage and remodeling. Neutrophil-mediated amplification was contact-dependent (Figure 4G). The amount of IL-12 produced in this experimental setting was sufficient to trigger IFNγ production by UTC_αβ_ but not by CD4^+^ and CD8^+^ conventional T cell populations isolated from the spleen of untreated control mice (Figure 4H). Importantly, upon exposure to relevant cytokines, sarcoma-infiltrating DNT_αβ_ cells are the most potent producers of IFNγ compared to other T cell subsets (Figure 4I). Collectively, these data suggest that the neutrophil-mediated maintenance of UTC_αβ_ type 1 polarization is driven by their higher sensitivity to IL-12 compared with other T cell populations.

### Single-Cell RNA-Seq Analysis of Tumor-Infiltrating UTC_αβ_

**Figure 5:**
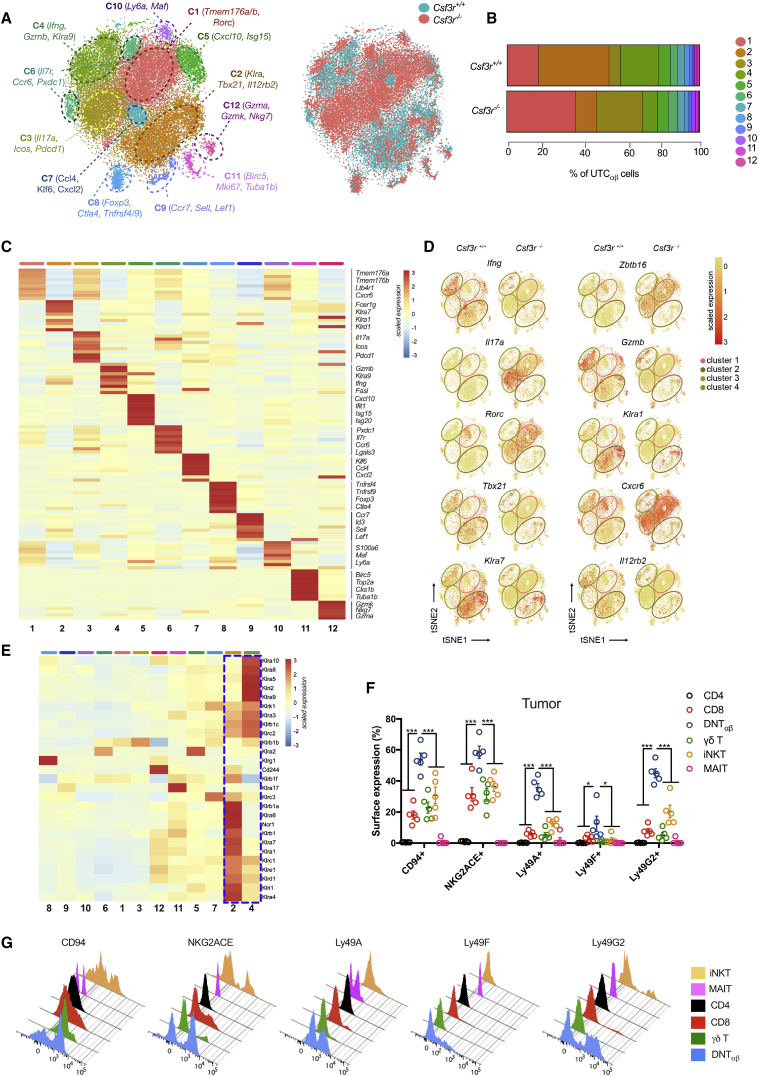
scRNA-Seq Analysis of Tumor-Infiltrating UTC_αβ_ (A) t-Distributed stochastic neighbor embedding (t-SNE) projection showing Seurat-guided unsupervised clustering and distribution of 31,623 UTC_αβ_ pooled from 2 *Csf3r*^+/+^ (14,721 cells) and 2 *Csf3r*^−/−^ (16,902 cells) sarcomas (see STAR Methods). Each point represents a single-cell colored according to cluster designation (left) or according to *Csf3r*^+/+^ and *Csf3r*^−/−^ conditions (right). (B) Bar graph showing the relative abundance of each cluster in *Csf3r*^+/+^ and *Csf3r*^−/−^ sarcomas, colored according to cluster designation. (C) Heatmap showing the top 10 differentially expressed genes in clusters 1–12. For each cluster, the average expression is plotted. Blue indicates lower expression, red indicates higher expression. Expression scale is shown on the right. (D) t-SNE projections showing the relative distribution of selected genes in *Csf3r*^+/+^ and *Csf3r*^−/−^ UTC_αβ_ cells. The position of clusters 1, 2, 3, and 4 are indicated. (E) Heatmap showing the expression of NK cell-related genes in sarcoma-infiltrating UTC_αβ_ cells. Clustering is based on their cluster-specific gene expression. A dotted blue line highlights the enrichment of NK cell-related genes in clusters 2 and 4. (F) Expression of NK cell-related molecules on *Csf3r*^+/+^ sarcoma-infiltrating T cell subsets as assessed by flow cytometry. (G) Representative histograms of the analysis shown in (F). (F) Data are mean ± SEM. ^∗∗∗^p ≤ 0.001. One-way ANOVA. See also Figures S5 and S6 and Table S4.

To dissect the diversity of tumor-associated UTC_αβ_ subsets, scRNA-seq was performed on sorted sarcoma-infiltrating UTC_αβ,_ isolated from *Csf3r*^+/+^ (14,721 cells) and *Csf3r*^−/−^ (16,902 cells) tumors. scRNA-seq analysis revealed the transcriptional complexity of the UTC_αβ_ population. Unsupervised clustering using Seurat methodology (Butler et al., 2018) allowed the identification of 12 clusters (Figures 5A and 5B).

Each cluster was characterized by a specific gene signature, associated to distinct effector functions, biological processes, and activation states (Figures 5A and 5C; Table S4). Clusters 1–4 represented more than 75% of total UTC_αβ_ and were differentially enriched in *Csf3r*^+/+^ and *Csf3r*^−/−^ sarcomas (Figures 5A and 5B). In particular, clusters 1 and 3 were enriched in *Csf3r*^−/−^ sarcomas (Figures 5A and 5B) and displayed pronounced expression of genes compatible with MAIT cell phenotype (e.g., *Cxcr6*, *Rorc*, *Icos*, *Zbtb16*) (Koay et al., 2016, Rahimpour et al., 2015) (Figures 5C and 5D; Table S4) and with the type 3 polarization state of Rorγt^+^ DNT_αβ_ cells (i.e., *Tmem176a-b*, *Il17a*, *Rorc)* (Figures 5C and 5D). On the other hand, clusters 2 and 4 were enriched in *Csf3r*^+/+^ sarcomas (Figures 5A and 5B) and presented high expression of effector molecules (i.e., *Gzmb*, *Ifng*, *Tbx21*) compatible with the type 1 activation state of T-bet^+^ DNT_αβ_ cells and genes related to Ly49 family (*Klra1*, *Klra7*, *Klra9*) (Figures 5C and 5D). The relative frequencies of cell subtypes obtained through flow cytometric analysis indicated a complete quantitative overlap of Rorγt^+^ DNT_αβ_ cells with cluster 1, MAIT cells with cluster 3, and T-bet^+^ DNT_αβ_ cells with clusters 2 and 4 (Figures S5H–S5J). Cluster 5 displayed a gene signature related to iNKT cells (Table S4), in line with quantitative data from flow cytometry analysis (Figure S5K). No expression of *Csf3r* was detected in any *Csf3r*^+/+^ UTC_αβ_ subset, thus excluding that lack of G-CSF-R signaling in UTC_αβ_ cells might impact on their polarization and function (data not shown).

**Figure S6:**
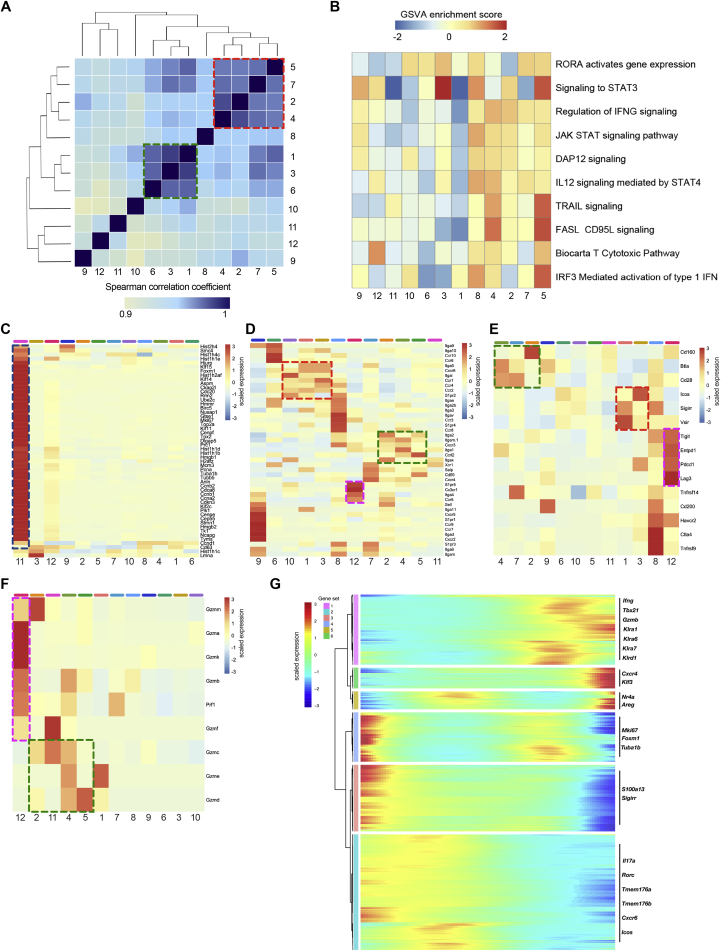
Functional Characterization of Sarcoma-Infiltrating UTC_αβ_ As Assessed by scRNA-Seq, Related to Figures 5 and 6 (A) Heatmap representing Spearman correlations among the twelve clusters identified, according to their transcriptional profiles. Green and red dashed boxes highlight the two functionally distinct cluster groups (red: type 1, abundant in *Csf3r*^+/+^ sarcomas; green: type 3, abundant in *Csf3r*^−/−^ sarcomas). (B) Heatmap showing GSVA enrichment score of selected pathways in clusters 1-12. Clusters are ordered according to their transcriptional similarity. (C-F) Heatmaps displaying expression of selected genes related to migration capacity (C), proliferation (D), checkpoint and costimulatory molecules (E) and cytolytic mediators (F). UTC_αβ_ cluster order is guided by gene expression. Green, red, blue and purple dashed boxes highlight the different functionally distinct cluster groups and are referred to in the main text. (G) Heatmap of differentially expressed genes, ordered according to their common expression variation through pseudotime (gene sets 1-6). Selected genes belonging to each gene set are highlighted on the right. (A-G) n = 2 mice per group. Scale bars showing Spearman correlation coefficient (A), GSVA enrichment score (B) or gene expression score (C-G) are provided. One experiment performed.

Correlation analysis performed on the whole transcriptome highlighted the existence of two main functionally distinct cluster groups (Figure S6A), which mirrored the respective composition of UTC_αβ_ subsets in *Csf3r*^+/+^ and *Csf3r*^−/−^ sarcomas. Gene set variation analysis (GSVA) performed on differentially expressed genes for each cluster showed the enrichment of IFNγ signaling and IL-12 signaling mediated by STAT4 in clusters enriched in *Csf3r*^+/+^ sarcomas (clusters 2 and 4) (Figure S6B), in line with data obtained by flow cytometry (Figure 3). Accordingly, *Il12rb2* expression was mainly confined to clusters 2 and 4 (Figure 5D), confirming their higher sensitivity to IL-12. These clusters were also characterized by enrichment in gene signatures associated with innate-like T cell activation and cytotoxic activity (e.g., DAP12 signaling, TRAIL signaling, FasL signaling, and T cytotoxic pathways) (Figure S6B).

To better characterize the 12 identified UTC_αβ_ clusters and their functional heterogeneity, we analyzed the gene expression of molecules related to key biological pathways. In particular, we assessed the enrichment of genes related to cell proliferation (Li et al., 2019) (Figure S6C), cell migration (Zhang et al., 2018) (Figure S6D), costimulatory molecules and immune checkpoints (Śledzińska et al., 2015), and effector molecules (Guo et al., 2018) (Figures S6E and S6F). Cluster 11 represented the only subset in active proliferation (Figure S6C). Clusters 1 and 3 were characterized by the expression of a specific set of immune checkpoints (i.e., *Icos*, *Sigirr*, and *Vsir*) and chemokine receptors (i.e., *Cxcr6*, *Ccr1*, and *Ccr4*). On the other hand, clusters 2 and 4 expressed several effector molecules (i.e., *Gzmb*, *Gzmd*, and *Prf1*) and a different set of chemokine receptors and costimulatory molecules, including *Cxcr3*, *Ccrl2*, and *Cd28* (Figures S6D–S6F). Among the other UTC_αβ_ subsets, cluster 12 expressed genes related to functionally active, terminally differentiated T cells (i.e., *Gzmk*, *Gzma*, *S1pr5*, *Cx3cr1*, and *Pdcd1*) (Figures S6D–S6F), while cluster 9 was characterized by the expression of migratory molecules typical of naive T cells such as *S1pr1*, *Ccr7*, and *Sell* (Figure S6D).

Importantly, clusters 2 and 4 showed a specific enrichment of NK cell-related genes, in particular those included in the Ly49 (*Klra*) and NKG2 (*Klrc*) receptor families (Figure 5E). We validated these findings by flow cytometry on sarcoma-infiltrating *Csf3r*^+/+^ T cells (Figures 5F and 5G).

Indeed, tumor-associated DNT_αβ_ cells displayed a unique set of Ly49 molecules (Figures 5F and 5G) and expressed higher levels of CD94 and NKG2ACE compared to any other T cell subset (Figures 5F and 5G).

**Figure 6:**
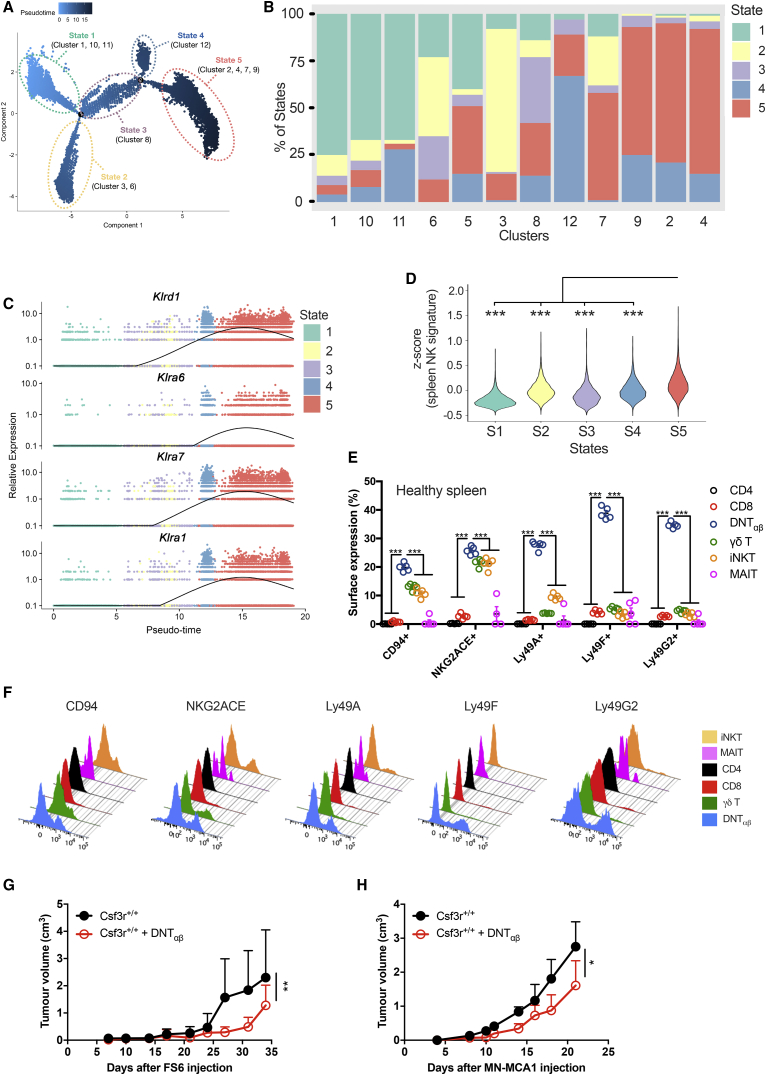
Ly49^+^ DNT_αβ_ Cells Display an Innate-like Phenotype and Antitumor Activity *In Vivo* (A) Monocle-guided cell trajectory orders five transcriptional states along an artificial pseudotime. Pseudotime is shown colored in a gradient from light to dark blue. (B) Analysis of UTC_αβ_ distribution along the transcriptional states described in (A). Bar graphs represent the frequency of cells belonging to each cluster in the five states. Clusters are ordered according to their calculated pseudotime mean score (from low to high pseudotime). (C) Representation of *Klra* gene expression plotted as a function of pseudotime. (D) Violin plots showing the enrichment of a splenic NK cell gene signature described in (Crinier et al., 2018) in the five transcriptional states identified by Monocle analysis. Mean *Z* score was calculated for each cell. Statistical significance was calculated comparing state 5 with every other state. (E) Expression of NK cell-related molecules on splenic *Csf3r*^+/+^ T cell subsets isolated from untreated mice, assessed by flow cytometry. (F) Representative histograms of the analysis shown in (D). (G and H) *In vivo* cotransfer assay was performed with sorted DNT_αβ_ cotransferred with two sarcoma cell lines. 10^5^ DNT_αβ_ were co-injected (subcutaneously [s.c.]) with either 2 × 10^6^ FS6 cells (G) or 5 × 10^5^ MN-MCA1 cells (H). (E) Data are mean ± SEM or (G and H) mean ± SD. ^∗^p ≤ 0.05, ^∗∗^p ≤ 0.01, ^∗∗∗^p ≤ 0.001. (D and E) One-way ANOVA. (G and H) Friedman test with Dunn’s multiple comparison test. See also Figure S5, Figure S6, Figure S7.

A Monocle-guided transcriptional trajectory identified five different functional states ordered along an artificial pseudotime, in which the 12 UTC_αβ_ clusters were differentially distributed (Figures 6A and 6B). The trajectory was defined by a gene set that included molecules related to T cell polarization (i.e., *Tbx21* and *Rorc*), effector functions (i.e., *Gzmb*, *Ifng*, and *Il17a*), and activation state (i.e., *Nr4a1*,*3*) (Figure S6G; Table S5). Interestingly, type 3 polarized cells (clusters 1 and 3) and type 1 polarized cells (clusters 2 and 4) were positioned at the opposite ends of the trajectory and represent the extremes in a spectrum of functional states (Figures 6A and S6G; Table S5). Importantly, the expression of several *Klra* genes was dynamically regulated during the trajectory and was higher in state 5 (Figures 6C and S6G). A previously validated splenic NK cell gene signature (Crinier et al., 2018) was significantly enriched in cells pertaining to state 5 (clusters 2 and 4), thus confirming their innate-like phenotype (Figure 6D). Notably, splenic DNT_αβ_ cells from untreated mice showed an array of receptors analogous to that observed in tumor-infiltrating DNT_αβ_ cells (Figures 6E and 6F), suggesting that the spleen might represent a Ly49^+^ DNT_αβ_ cell reservoir. Thus, these results provide insight into the diversity of tumor-associated UTC_αβ_ at a single-cell level and highlight a subset of Ly49R-expressing UTC_αβ_ (i.e., DNT_αβ_ in clusters 2 and 4) with type 1 polarization and potential antitumor activity.

In an effort to obtain an indication as to the actual antitumor potential of DNT_αβ_ cells, we conducted an *in vivo* cotransfer model in two transplantable murine sarcoma models (MN-MCA1 and FS6) (Bonavita et al., 2015). At low DNT_αβ_/tumor cell ratios (1:5 and 1:20), DNT_αβ_ cells significantly reduced tumor growth (Figure 6G and 6H). Thus, DNT_αβ_ cells can indeed mediate antitumor resistance *in vivo*.

### Neutrophil Infiltration Is Associated with Better Prognosis and Type 1 Immunity in Selected Human Tumors

**Figure 7:**
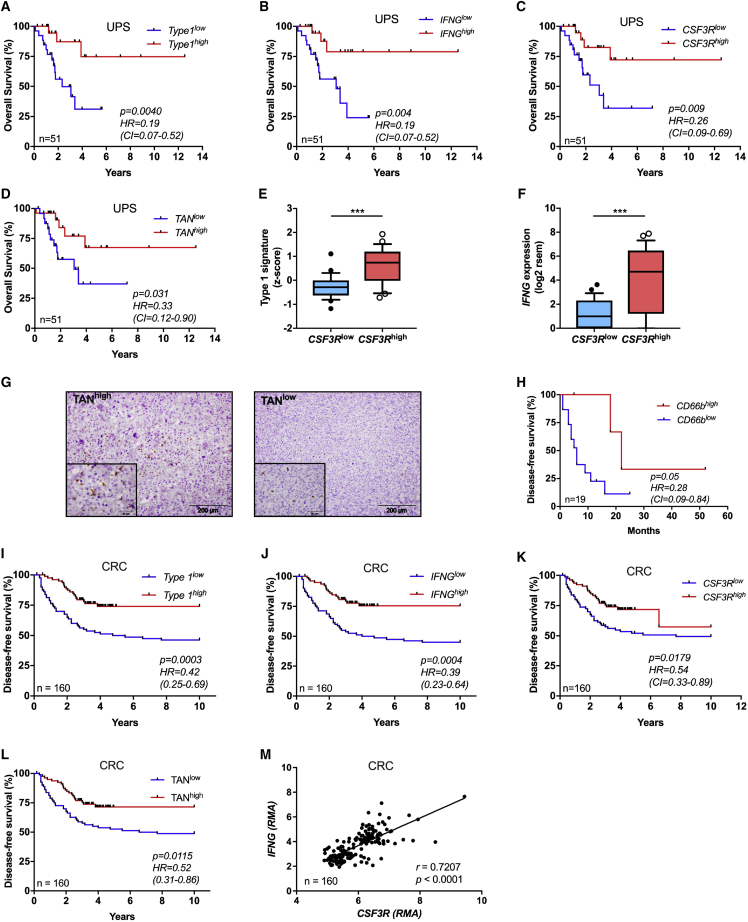
Neutrophils Are Associated With Better Prognosis and Type 1 Immune Response in Human Undifferentiated UPS and Colorectal Cancer (A) Kaplan-Meier survival curve of UPS patients from TCGA cohort with respect to high or low expression of 12 genes related to type 1 signature within tumor specimens. (B) Kaplan-Meier survival curve of UPS patients from TCGA cohort with high or low expression of *IFNG* within tumor specimens. (C and D) Kaplan-Meier survival curves of UPS patients from TCGA cohort with high or low expression of *CSF3R* (C) or TAN gene signature (31 genes) (D) within tumor specimens. (E and F) Relative expression of type 1 gene signature (E) and *IFNG* (F) in tumor samples of UPS patients from TCGA cohort. Patients were divided in two groups according to their high or low expression of *CSF3R*. (G) Example of high and low neutrophil infiltration in human UPS assessed by histological analysis of paraffin-embedded UPS sarcoma samples from Humanitas Clinical and Research Center stained with anti-CD66b antibody. (H) Kaplan-Meier curve shows recurrence-free survival for UPS patients from Humanitas Clinical and Research Center presenting a high or low TAN infiltration (CD66b^high^ and CD66b^low^ assessed by immunohistochemistry). (I–L) Kaplan-Meier survival curves of CRC patients stratified according to their type 1 gene signature (I), *IFNG* (J), *CSF3R* (K), or neutrophil gene signature expression. (M) Pearson correlation between *CSF3R* and *IFNG* expression in CRC patients. Gene expression is reported as robust multi-array average (RMA). (A–D and H–M) Numbers depicted in each graph represent the total number of patients analyzed. (E and F) Boxes: 25–75 range; whiskers: 10–90 range. (E and F) ^∗∗∗^p ≤ 0.001. (A–D and H–L) log-rank (Mantel-Cox) test. (E and F) Two-tailed Mann-Whitney U-test; HR, hazard ratio; CI, 95% confidence interval. (M) Pearson correlation. See also Figure S7 and Tables S6 and S7.

**Figure S7:**
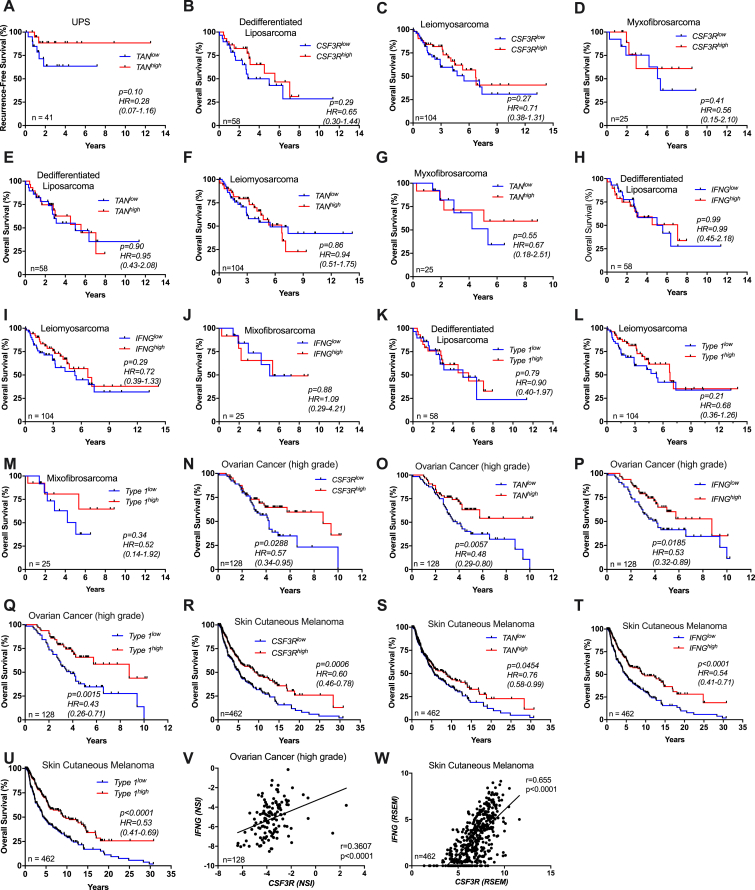
Clinical Significance of TAN Infiltration and Type 1 Immune Response in Soft Tissue Sarcoma Subtypes and Selected Human Tumors, Related to Figure 7 (A) Kaplan-Meier curve of recurrence-free survival in UPS patients from TCGA cohort. (B-M) Kaplan-Meier overall survival curves of patients from TCGA cohort with dedifferentiated liposarcoma, leiomyosarcoma and myxofibrosarcoma with high or low expression of (B-D) *CSF3R*, (E-G) TAN gene signature (31 genes), (H-J) *IFNG* and (K-M) 12 genes related to type 1 immune response within tumor specimens. (N-U) Kaplan-Meier survival curves of patients of ovarian cancer (N-Q) and skin cutaneous melanoma (R-U) with high or low expression of *CSF3R*, TAN gene signature, *IFNG* and type 1 immune gene signature within tumor specimens. (V-W) Pearson correlation between *CSF3R* and *IFNG* expression in ovarian cancer and melanoma patients. Gene expression is reported as NSI or as RSEM, respectively (see Methods). Gene lists used for type 1 immune response and neutrophil signatures are shown in Table S6. (A-W) Numbers depicted in each graph represent the total number of patients analyzed. (A-U) *p* and HR, Hazard Ratio (95% CI ratio) were calculated with log-rank (Mantel-Cox) test. (V-W) Pearson correlation.

The results reported above identify a novel neutrophil-orchestrated pathway of effective type 1 immunity against sarcomagenesis. It was therefore important to explore its significance in human disease. Human soft tissue sarcomas (STS) are a heterogeneous and complex set of neoplasias in terms of genetic abnormalities and clinical behavior, responsible for ∼5,000 deaths per year in the United States (American Cancer Society, 2017, Taylor et al., 2011). We interrogated the RNA-seq The Cancer Genome Atlas (TCGA) database and found that in undifferentiated UPS a type 1 immune response gene signature and *IFN*□ were associated with favorable outcome (Figures 7A and 7B; Table S6). Moreover, *CSF3R* expression was also associated with better outcome in terms of overall survival in UPS patients (Figure 7C). Using a previously validated neutrophil-specific gene signature (Bindea et al., 2013, Chao et al., 2016) (31 genes, listed in Table S6), UPS patients were divided into TAN^high^ and TAN^low^ subgroups. The resulting Kaplan-Meier curve showed that patients with TAN^high^ tumor biopsies at diagnosis had a significant survival advantage compared to TAN^low^ patients and a trend was observed for recurrence-free survival (hazard ratio [HR] 0.28; 95% confidence interval [CI] 0.07–1.16) (Figures 7D and S7A). Interestingly, high *CSF3R* expression was associated with a type 1 immunity signature and with *IFNG* expression (Figures 7E and 7F). No association was observed between *CSF3R*, neutrophil signature, *IFNG*, or type 1 immune signature and outcome in other sarcomas (i.e., dedifferentiated liposarcoma, leiomyosarcoma and myxofibrosarcoma) (Figures S7B–S7M). TAN infiltration in human UPS tumor specimens was validated by immunohistochemistry for CD66b in a separate cohort of 19 UPS patients followed at Humanitas Clinical and Research Center (Figure 7G; Table S7). The mean number of neutrophil infiltration ranged from 1 to 17 cells per field (Figure 7G). Recurrence-free survival was higher in CD66b^high^ UPS patients (Figure 7H). Interestingly UPS, which accounts for 14% of total STS (Brennan et al., 2014), has been suggested to be the counterpart of 3-MCA-induced sarcomas (Katenkamp et al., 1988, Li et al., 2010).

We extended our analysis to other human cancer datasets and found a significantly positive correlation between the neutrophilic infiltrate, *IFNG* expression, a type 1 immune response gene signature and better prognosis in colorectal cancer (CRC) (Figures 7I–7L). Moreover, *CSF3R* was positively correlated with *IFNG* expression (Figure 7M), suggesting that the neutrophil-IFNγ axis might be relevant in selected human tumors. Interestingly, high neutrophil infiltration assessed by immunohistochemistry has previously been associated with better outcome in CRC in five independent reports (Berry et al., 2017, Bindea et al., 2013, Galdiero et al., 2016, Governa et al., 2017, Wikberg et al., 2017). These results suggest that, mirroring findings in 3-MCA carcinogenesis, a neutrophil-type 1 immunity axis may play a role in resistance against selected human tumors (Figures 7 and S7N–S7W), in particular UPS and CRC.

## Discussion

Evidence based on antibody-mediated depletion and on clinical associations suggests that neutrophils can exert a dual influence on carcinogenesis, progression to metastasis and response to therapy (Coffelt et al., 2016, Engblom et al., 2017, Fridlender et al., 2009, Galdiero et al., 2016, Granot et al., 2011, Massara et al., 2018, Wculek and Malanchi, 2015). Here, we provide unequivocal genetic evidence based on *Csf3r* deficiency, supported by antibody-dependent depletion and adoptive cell transfer, that neutrophils are essential for mounting an effective type 1 IFNγ-dependent immune response, which restrains 3-MCA sarcomagenesis.

T cells and IFNγ have long been known to mediate resistance against 3-MCA-driven carcinogenesis (Kaplan et al., 1998, Koebel et al., 2007, Shankaran et al., 2001). Here, we report that neutrophil deficiency was associated with a selective functional skewing of UTC_αβ_ cells with no discernable impact on the polarization state of other T cell subsets. It remains unclear whether the enhanced carcinogenesis observed in *Csf3r*^−/−^ neutrophil-deficient mice is only a reflection of defective IFNγ production, increased skewing to a type 3 IL-17 response, or a combination of the two. The discovery of a neutrophil/UTC_αβ_ axis, relevant to the control of mesenchymal carcinogenesis, raises the general issue of the relevance of innate-like UTC_αβ_ cells in cancer.

The neutrophil-dependent pathway of resistance to sarcoma induction by 3-MCA involved IL-12 produced by macrophages. In an *in vitro* coculture model, neutrophils dramatically amplified macrophage-derived IL-12 release, which was sufficient to trigger IFNγ production by UTC_αβ_ but not by CD4^+^ or CD8^+^ T cells. This finding is reminiscent of a previously reported innate lymphoid cell subset, characterized by a unique capacity to produce IFNγ in response to IL-12 (Fuchs et al., 2013). Thus, in 3-MCA sarcomagenesis a tripartite interaction involving neutrophils, macrophages, and UTC_αβ_ is an essential component of type 1 immune resistance.

scRNA-seq analysis showed that tumor infiltrating UTC_αβ_ were highly heterogeneous. Twelve clusters were identified, and four of them represented over 75% of the total UTC_αβ_ pool. These clusters included cells with molecular signatures of MAIT cells. *Csf3r* deficiency was associated with selective depletion of DNT_αβ_ cells belonging to clusters 2 and 4, characterized by a gene expression repertoire indicative of antitumor effector function. These cells expressed several NK cell-related molecules, such as Ly49 inhibitory receptors as well as CD94/NKG2A inhibitory complex. Ly49^+^ DNT_αβ_ cells were present in the spleen of untreated mice and were able to mediate antitumor activity *in vivo* suggesting that the spleen might represent a reservoir of Ly49^+^ DNT_αβ_ cells endowed with antitumor potential. Interestingly, the targets of current checkpoint blockade therapies PD-1 and CTLA-4 were not prominently expressed by these cells. Thus, the present results suggest that in tumors in which there is evidence for a neutrophil/IFNγ resistance pathway, targeting DNT_αβ_ cell inhibitory receptors should be considered as an alternative or complementary strategy. These results highlight for the first time the presence, diversity, and antitumor potential of UTC_αβ_ in the TME and suggest that neutrophils can sustain the antitumor potential of Ly49^+^ DNT_αβ_ cells.

A type 1 immune response signature, *IFNG*, *CSF3R*, and a neutrophil signature were associated with better survival in selected human tumors, including UPS and CRC (Figures 7 and S7N–S7W). The finding of neutrophil infiltration being associated with better outcome based on *in silico* analyses of public databases was consistent with data obtained by immunohistochemistry for UPS in the present report (Figures 7G and 7H) and for CRC in five independent previous studies with large case lists (Berry et al., 2017, Bindea et al., 2013, Galdiero et al., 2016, Governa et al., 2017, Wikberg et al., 2017). These results strongly suggest that the neutrophil-dependent pathway of antitumor resistance described in 3-MCA carcinogenesis is indeed relevant in selected human tumors. This neutrophil-type 1 immunity axis may have broader significance in neoplastic and non-neoplastic conditions.

The results reported here emphasize the diversity of mechanisms of immune resistance in human tumors, even when histologically related, and call for tailoring of immunotherapy strategies and correlate biomarkers, including neutrophil-related ones, to the diversity of immune pathways. Moreover, the occurrence and significance of UTC_αβ_ in the TME may have been underestimated.

## STAR★Methods

### Key Resources Table

**Table:** 

REAGENT or RESOURCE	SOURCE	IDENTIFIER
**Antibodies and tetramers**		

CD103-PerCPeF710 (2E7)	eBioscience	Cat # 46103182; RRID:AB_2573704
CD11b-BV421 (M1/70)	BioLegend	Cat # 101236; RRID: AB_11203704
CD11b-BV480 (M1/70)	BD Biosciences	Cat # 5666117; RRID:AB_2739519
CD11b-BV786 (M1/70)	BioLegend	Cat # 101243; RRID:AB_2561373
CD11b-APCCy7 (M1/70)	BD Biosciences	Cat # 557657; RRID:AB_396772
CD11b-FITC (M1/70)	BioLegend	Cat # 101206; RRID:AB_312789
CD11c-PE (HL3)	BD Biosciences	Cat # 553802; RRID:AB_395061
CD11c-AlexaFluor700 (HL3)	BD Biosciences	Cat # 560583; RRID:AB_1727421
CD19-PE (1D3)	BD Biosciences	Cat # 553786; RRID:AB_395050
CD19-PerCPCy5.5 (1D3)	BD Biosciences	Cat # 551001; RRID:AB_394004
CD19-eFluor450 (1D3)	eBioscience	Cat # 48019382; RRID:AB_2734905
CD24-APCeFluor780 (M1/69)	eBioscience	Cat # 47024282; RRID:AB_10853172
CD27-APCeFluor780 (LG.7F9)	eBioscience	Cat # 47027180; RRID:AB_10854880
CD27-PECy7 (LG.7F9)	eBioscience	Cat # 25027182; RRID:AB_1724035
CD27-FITC (LG.7F9)	eBioscience	Cat # 11027185; RRID:AB_465002
CD3e-APC (145-2C11)	eBioscience	Cat # 17003182; RRID:AB_469315
CD3e-PerCP-Cy5.5 (145-2C11)	eBioscience	Cat # 45003182; RRID:AB_1107000
CD4-AlexaFluor 700 (RM4-5)	BD Biosciences	Cat # 557956; RRID:AB_396956
CD4-FITC (H129.19)	BioLegend	Cat # 130308; RRID:AB_1279237
CD45-BV605 (30-F11)	BD Biosciences	Cat # 563053; RRID:AB_2737976
CD45-BV650 (30-F11)	BD Biosciences	Cat # 563410; RRID:AB_2738189
CD45-PerCP-Cy5.5 (30-F11)	eBioscience	Cat # 45045182; RRID:AB_1107002
CD45.2-BUV805 (104)	BD Biosciences	Cat # 741957; RRID: NA
CD49a-BV711 (Ha31/8)	BD Biosciences	Cat # 564863; RRID:AB_2738987
CD49b-APC (DX5)	eBioscience	Cat # 17597182; RRID:AB_469485
CD54-PE (YN1/1.7.4)	BioLegend	Cat # 116108; RRID:AB_313699
CD62L-APC (MEL-14)	BD Biosciences	Cat # 553152; RRID:AB_398533
CD62L-BV570 (MEL-14)	BioLegend	Cat # 104433; RRID:AB_10900262
CD64-PE (X54-5/7.1)	BioLegend	Cat # 139304; RRID:AB_10612740
CD86-eFluor450 (GL-1)	eBioscience	Cat # 48086280; RRID:AB_2574030
CD8a-BV480 (53-6.7)	BD Biosciences	Cat # 566096; RRID:AB_2739566
CD8a-BV570(53-6.7)	BioLegend	Cat # 100740; RRID:AB_2563055
CD8a-PE (53-6.7)	Invitrogen	Cat # 12008182; RRID:AB_465530
CD94-BV650 (18d3)	BD Biosciences	Cat # 740551; RRID:AB_2740252
F4/80-PECy7 (BM8)	BioLegend	Cat # 123114; RRID:AB_893478
KLRG1-BV786 (2F1)	BD Biosciences	Cat # 565477; RRID:AB_2739256
Ly49A-BUV395 (A1)	BD Biosciences	Cat # 742370; RRID:AB_2740728
Ly49C-BV605 (5E6)	BD Biosciences	Cat # 744029; RRID:AB_2741939
Ly49F-BV421 (HBF-719)	BD Biosciences	Cat # 744777; RRID:AB_2742475
Ly49G2-BV480 (4D11)	BD Biosciences	Cat # 746794; RRID:AB_2741127
Ly6C-BV421 (AL21)	BD Biosciences	Cat # 562727; RRID:AB_2737748
Ly6C-FITC (AL21)	BD Biosciences	Cat # 561085; RRID:AB_10584332
Ly6G-BUV395 (1A8)	BD Biosciences	Cat # 563978; RRID:AB_2716852
Ly6G-PECF594 (1A8)	BD Biosciences	Cat # 562700; RRID:AB_2737730
MHCII-BV711 (2G9)	BD Biosciences	Cat # 743874; RRID:AB_2741825
MHCII-FITC (2G9)	BD Biosciences	Cat # 553623; RRID:AB_394958
MHCII-PerCP (M5/114.15.2)	BD Biosciences	Cat # 562363; RRID:AB_562363
NK1.1-PE (PK136)	eBioscience	Cat # 12594182; RRID:AB_466050
NK1.1-APC (PK136)	eBioscience	Cat # 17594182; RRID:AB_469479
NK1.1-BV650 (PK136)	BD Biosciences	Cat # 564143; RRID:AB_564143
NK1.1-PECF594 (PK136)	BD Biosciences	Cat # 562864; RRID:AB_2737850
NKG2A/C/E-BUV563 (18d3)	BD Biosciences	Cat # 741339; RRID:AB_741339
NKp46-BV421 (29A1.4)	BioLegend	Cat # 137612; RRID:AB_2563104
TCRβ-BV711 (H57-597)	BD Biosciences	Cat # 563135; RRID:AB_2738023
TCRγδ-BV421 (GL3)	BD Biosciences	Cat # 562892; RRID:AB_2737871
TCRγδ-PerCPeFluor710 (GL3)	eBioscience	Cat # 46571182; RRID:AB_2016707
Eomes-AlexaFluor488 (Dan11Mag)	eBioscience	Cat # 53487582; RRID:AB_10854265
IFNγ-AlexaFluor700 (XMG1.2)	BD Biosciences	Cat # 557998; RRID:AB_396979
IFNγ-BV421 (XMG1.2)	BD Biosciences	Cat # 563376; RRID:AB_2744290
PLZF-AlexaFluor647 (R17-809)	BD Biosciences	Cat # 563490; RRID:AB_563490
RORγT-PECF594 (Q31-378)	BD Biosciences	Cat # 562684; RRID:AB_2651150
Tbet-PE (O4-46)	BD Biosciences	Cat # 561268; RRID:AB_10564071
Tbet-BV780 (O4-46)	BD Biosciences	Cat # 564141; RRID:AB_2738615
αGalCer-CD1d-APC	ProImmune	Cat # E001-4B-E; RRID:NA
5-OP-RU-MR1-BV421	James McCluskey, University of Melbourne	N/A
5-OP-RU-MR1-PE	James McCluskey, University of Melbourne	N/A
Rat anti-Ly6G (1A8)	BioXCell	Cat # BP0075-1; RRID:AB_1107721
Rat anti-iFNγ (XMG1.2)	BioXCell	Cat # BE0055; RRID:AB_1107694
Rat anti-iL-12p75 (R2-9A5)	BioXCell	Cat # BE0233; RRID:AB_2687715
Rat anti-CD115 (AFS98)	BioXCell	Cat # BE0213; RRID:AB_2687699
Rat Isotype Control (2A3)	BioXCell	Cat # BE0089; RRID:AB_1107769
Rat Isotype Control (LTF-2)	BioXCell	Cat # BE0090; RRID:AB_1107780
Rat Isotype Control (HRPN)	BioXCell	Cat # BE0088; RRID:AB_1107775
Rat Anti-mouse CD31 (MEC13.3)	BD Bioscences	Cat # 553370; RRID:AB_394816
Mouse Anti-human CD66b (G10F5)	BD Bioscences	Cat # 555723; RRID:AB_396066

**Biological Samples**		

Surgical Samples from UPS patients	Humanitas Clinical&Research Hospital	N/A

**Chemicals, Peptides, and Recombinant Proteins**		

3-Methylcolanthrene	Sigma Aldrich	Cat # 213942
Recombinant mouse GM-CSF	Peprotech	Cat # 315-03
Recombinant mouse G-CSF	Peprotech	Cat # 250-05
Recombinant mouse M-CSF	Peprotech	Cat # 315-02
Recombinant mouse IFNγ	Peprotech	Cat # 315-05
Recombinant mouse IL-4	Peprotech	Cat # 214-14
Recombinant mouse IL-12	Peprotech	Cat # 210-12
Recombinant human/mouse IL18	MBL	Cat # B001-5
Proleukin, IL-2	Novartis	N/A
ODN 1826 Murine TLR9 Ligand (CpG)	InvivoGen	Cat # Tlrl-1826
cAIMP Difluor	InvivoGen	Cat # Tlrl-nacaidf
Collagenase from *Clostridium Histolyticum*	Sigma Aldrich	Cat # C5138
Liberase TM	Roche	Cat # 541119001
DNase I	Roche	Cat # 4536282001

**Critical Commercial Assays**		

Live/Dead fixable Dye Aqua Cell Dead stain kit, 405nm	Invitrogen	Cat # L34957
Live/Dead fixable Dye eFluor780	eBioscience	Cat # 65-0865-18
Foxp3 Staining Buffer Set	eBioscience	Cat # 00-5523-00
BD GolgiPlug	BD Biosciences	Cat # 555029
Cell Stimulation Cocktail	eBioscience	Cat # 00-4970-03
CD16/CD32 FcBlock (24G2)	eBioscience	Cat # 16016185
Rat Serum	eBioscience	Cat # 24555594
Click-iT EdU AlexaFluor 647 Flow Cytometry Assay Kit	Invitrogen	Cat # C10419
PrimeFlow RNA Assay Kit	Thermo Fisher	Cat # 88-18005-210
*Mus musculus Il12b* (RUO) Probe	Thermo Fisher	Cat # VB4-20451
*Mus musculus Il12a* (RUO) Probe	Thermo Fisher	Cat # VB1-12422
*Mus musculus Actb* (RUO) Probe	Thermo Fisher	Cat # VB1-10350
Chromium Single Cell 3′ Library and Gel Bead Kit v2, 4 rxns	10X Genomics	Cat # FC5120267
Chromium Single Cell A Chip Kit, 16 rxns	10X Genomics	Cat # FC51000009
NSQ500/550 Hi Output KT v2.5 (75CYS)	Illumina	Cat # 20024906
NSQ 500 hi-Output RGT CART v2 (75CYS)	Illumina	Cat # 15057934
NextSeq High Output Flow Cell v2.5	Illumina	Cat # 20022408
NextSeq 500/550 Buffer Cartridge v2	Illumina	Cat # 15057941
NextSeq Accessory Box v2	Illumina	Cat # 15058251
Neutrophil Isolation Kit, mouse	Miltenyi Biotec	Cat # 130-097-658
Pan T cell Isolation Kit II, mouse	Miltenyi Biotec	Cat # 130-095-130
CD45 (TIL) MicroBeads, mouse	Miltenyi Biotec	Cat # 130-110-618
CD4^+^/CD8^+^ (TIL) Microbeads, mouse	Miltenyi Biotec	Cat # 130-116-480
CD11b^+^ Microbeads, human and mouse	Miltenyi Biotec	Cat # 130-049-601
RNeasy MinElute Cleanup Kit	QIAGEN	Cat # 74204
Maxwell^®^ 16 LEV simplyRNA Cell Kit	Promega	Cat # AS1270
High-Capacity cDNA Reverse Transcription Kit	Applied Biosystems	Cat # 4368814
SYBR™ Green PCR Master Mix	Applied Biosystems	Cat # 4309155

**Deposited Data**		

Raw and processed bulk RNaseq data	NCBI GEO	GSE109031
Raw and processed single cell RNaseq data	NCBI GEO	GSE123508

**Experimental Models: Cell Lines**		

MN-MCA1	[Bibr bib51][Bibr bib5]	N/A
FS6	Mantovani et al.,1977	N/A

**Experimental Models: Organisms/Strains**		

*Csf3r*^−/−^	Jackson Laboratory	Cat # 017838
C57BL/6J	Jackson Laboratory	Cat # 000664

**Oligonucleotides**		

*Retnla* (*Fizz1*): forward 5′-CCC TTC TCA TCT GCA TCT CC-3′ reverse 5′-CTG GAT TGG CAA GAA GTT CC-3′	This Paper	N/A
*Chil3* (*Ym1*): forward 5′-TCT GGG TAC AAG ATC CCT GAA-3′ reverse 5′-TTT CTC CAG TGT AGC CAT CCT T-3′	This Paper	N/A
*Ccl5*: forward 5′-TGC AGA GGA CTC TGA GAC AGC-3′ reverse 5′-GAG TGG TGT CCG AGC CAT A-3′	This Paper	N/A
*Nos2*: forward 5′-GCC ACC AAC AAT GGC AAT A-3′ reverse 5′-CGT ACC GGA TGA GCT GTG AAT T-3′	This Paper	N/A
*Ccl2*: forward 5′- ATT GGG ATC ATC TTG CTG GT-3′ reverse 5′- CCT GCT GTT CAC AGT TGC C-3′	This Paper	N/A
*Arg1*: forward 5′- TTT TTC CAG CAG ACC AGC TT-3′ reverse 5′- AGA GAT TAT CGG AGC GCC TT-3′	This Paper	N/A
*Tgfb1*: forward 5′-CAA CCC AGG TCC TTC CTA AA-3′ reverse 5′- GGA GAG CCC TGG ATA CCA AC-3′	This Paper	N/A
*Il27p28*: forward 5′- AGC TCT TGA AGG CTC AGG G-3′ reverse 5′- GTG ACA GGA GAC CTT GGC TG-3′	This Paper	N/A
*Ifng*: forward 5′-TCA AGT GGC ATA GAT GTG GAA GAA-3′ reverse 5′-TGG CTC TGC AGG ATT TTC ATG-3′	This Paper	N/A
*Il10*: forward 5′-GGT TGC CAA GCC TTA TCG GA-3′ reverse 5′-ACC TGC TCC ACT GCC TTG CT-3′	This Paper	N/A
*Stab1*: forward 5′-CCC TCC TTC TGC TCT GTG TC-3′ reverse 5′- CAA ACT TGG TGT GGA TGT CG-3′	This Paper	N/A
*Mrc1*: forward 5′-TGG CAT GTC CTG GAA TGA T-3′ reverse 5′-CAG GTG TGG GCT CAG GTA GT-3′	This Paper	N/A
*Msr1*: forward 5′-TGC CCT CAT TGC TCT CTA CC-3′ reverse 5′- TTC ATT TCC CAA TTC AAA AGC TC-3′	This Paper	N/A
*Il4ra*: forward 5′- GTG GAG CCT GAA CTC GCA-3′ reverse 5′-AAG CAC GCA GAT CCA AAA TC-3′	This Paper	N/A
*Marco*: forward 5′-TTC TGT CGC ATG CTC GGT TA-3′ reverse 5′-CAG ATG TTC CCA GAG CCA CC-3′	This Paper	N/A
*Met*: forward 5′-TGT CCG ATA CTC GTC ACT GC-3′ reverse 5′-CAT TTT TAC GGA CCC AAC CA-3′	This Paper	N/A
*Il1b*: forward 5′- GGT CAA AGG TTT GGA AGC AG-3′ reverse 5′- TGT GAA ATG CCA CCT TTT GA-3′	This Paper	N/A
*Ccl3*: forward 5′- GTGGAATCTTCCGGCTGTAG-3′ reverse 5′- ACCATGACACTCTGCAACCA-3′	This Paper	N/A
*Cxcl10*: forward 5′-CCG TCA TTT TCT CCC TCA TCC-3′ reverse 5′- CCC TAT GGC TGC TTC ACT CTC A-3′	This Paper	N/A
*Il23a*: forward 5′-AGC ATT TAT GCT TCC AAA GC-3′ reverse 5′-GGA GGT CTC AAG TTC CTA CAT G-3′	This Paper	N/A
*Ccr3*: forward 5′-TGA AAC TGT GAT CTT GGG ACA-3′ reverse 5′-CAG CAT CAA CAA CAC GTT CC-3′	This Paper	N/A
*Il1r5*: forward 5′-GAT GCA TGT TTA GGC TTC CA-3′ reverse 5′-TCT TCT GCT GTC TGG AGC AA-3′	This Paper	N/A
*Il1r7*: forward 5′-AGA GTG CAG AGA GGC AAA CC-3′ reverse 5′-TGA AAC CCT CTT CCT CCA GA-3′	This Paper	N/A
*Il12rb1*: forward 5′- GCA GCC GAG TGA TGT ACA AG-3′ reverse 5′- GAG ACG CGA AAA TGA TGG AT-3	This Paper	N/A
*Il12rb2*: forward 5′- AAC ACC AGA GGA AGA GCC TG-3′ reverse 5′- CGT CAC CTG ATA GTG GAG GA-3′	This Paper	N/A
*Gapdh/GAPDH*: forward 5′-GCA AAG TGG AGA TTG TTG CCA T-3′ reverse 5′-CCT TGA CTG TGC CGT TGA ATT T-3′	This Paper	N/A

**Software and Algorithms**		

FlowJo v9.3	FlowJo	https://www.flowjo.com/solutions/flowjo/downloads
Prism v7	GraphPad	https://www.graphpad.com
FastQC (v.0.11.6)	FastQC	http://www.bioinformatics.babraham.ac.uk/projects/fastqc
STAR (v.020201)	Dobin et al., 2013	https://github.com/alexdobin/STAR
edgeR (v.3.24.1)	Robinson et al., 2010	http://bioconductor.org/packages/release/bioc/html/edgeR.html
CellRanger (v.2.1.1)	10X Genomics	https://support.10xgenomics.com/single-cell-gene-expression/software/pipelines/latest/what-is-cell-ranger
Seurat (v.2.3.4)	Butler et al., 2018	https://satijalab.org/seurat/
Monocle (v.2.8.0)	Trapnell et al., 2014, Qiu et al., 2017	http://cole-trapnell-lab.github.io/monocle-release/

### Contact for Reagent and Resource Sharing

Further information and requests for resources and reagents should be directed to and will be fulfilled by the Lead Contact, Alberto Mantovani (alberto.mantovani@humanitasresearch.it) or to Sebastien Jaillon (sebastien.jaillon@humanitasresearch.it).

### Experimental Model and Subject Details

#### Animals

All mice used were on a C57BL/6J genetic background. *Csf3r*-deficient mice were purchased from the Jackson Laboratory, Bar Harbour, Maine, US. All colonies were housed and bred in the SPF animal facility of Humanitas Clinical and Research Center in individually ventilated cages. Mice were randomized based on age and weight.

Procedures involving animals handling and care were conformed to protocols approved by the Humanitas Clinical and Research Center (Rozzano, Milan, Italy) in compliance with national (D.L. N.116, G.U., suppl. 40, 18-2-1992 and N. 26, G.U. March 4, 2014) and international law and policies (EEC Council Directive 2010/63/EU, OJ L 276/33, 22-09-2010; National Institutes of Health Guide for the Care and Use of Laboratory Animals, US National Research Council, 2011). The study was approved by the Italian Ministry of Health (approvals n. 334/2013-B, issued on 27/12/2013 and n. 261/2017-PR issued on 28/03/2017). All efforts were made to minimize the number of animals used and their suffering. In most *in vivo* experiments, the investigators were unaware of the genotype of the experimental groups.

#### Carcinogen-induced sarcoma model

Male mice were injected s.c. with a single dose of 100 μg of 3-MCA (Sigma-Aldrich, St Louis, US) dissolved in corn oil and assessed for tumor development over the course of 5 months. Data are shown as tumor incidence and tumor volume according to the formula: Volume = (Dxd^2^)/2, where D = larger tumor diameter and d = smaller tumor diameter, during the entire duration of the experiment using a calliper. In neutrophil adoptive transfer experiments, tumor growth is expressed over time (days) after the first palpable tumor observation (V = 4 mm^3^). In cohousing experiments, *Csf3r*^+/+^ and *Csf3r*^−/−^ male mice were cohoused in a 1:1 ratio from birth until 8 weeks of age, and then injected with 3-MCA.

#### Depletion and blocking experiments

For neutrophil and IFNγ depletions, mice were intraperitoneally (i.p.) treated with 200 μg of specific mAbs (Rat anti-Ly6G, Clone 1A8; Rat Isotype Control Clone 2A3; Rat anti-iFNγ, Clone XMG1.2; Rat Isotype Control, Clone HRPN) on the day before 3-MCA administration, and with 100 μg twice a week for the entire duration of the experiment. In IL-12p75 depletion experiments, mice were i.p. treated with 1 mg of Rat anti-iL-12p75 (Clone R2-9A5; Rat Isotype Control, Clone LTF-2) on the day before 3-MCA administration, and with 500 μg on days +1 and +6 after 3-MCA administration. For M-CSFR blockade experiments mice were i.p. treated as previously shown (Hashimoto et al., 2011) with 1.5 mg of Rat anti-CD115 (Clone AFS98; Rat Isotype Control, Clone 2A3) on the day before 3-MCA administration, and with 300 μg twice a week after 3-MCA administration until mice reached experimental endpoint. In a second set of experiment, mice were i.p. treated with 1.5 mg on the day before 3-MCA administration and with 300 μg on days 0, +3 and +7 after 3-MCA administration. All depleting antibodies were purchased from Bioxcell (West Lebanon, US).

#### Neutrophil adoptive transfer

In a first set of experiments, 3x10^6^ MACS-enriched bone marrow neutrophils (Purity ≥ 97.5%) isolated from naive *Csf3r*^+/+^ mice were injected i.v. in *Csf3r*^−/−^ sarcoma-bearing mice, once a week, starting from the first visible observation of tumor occurrence (range 75-100 days after 3-MCA administration), until mice reached the experimental endpoint. In a second set of experiments, neutrophils were injected in *Csf3r*^−/−^ mice on day −1, 0, 1 and 9 with respect to 3-MCA administration, and mice were sacrificed on day 10.

#### Generation of Bone Marrow Chimeras

*Csf3r*-competent and -deficient mice were lethally irradiated with a total dose of 900 cGy. 2 hours later mice were injected in the retro-orbital plexus with 4x10^6^ nucleated bone marrow cells obtained by flushing of the cavity of a freshly dissected femur from wild-type or *Csf3r*-deficient donors. Recipient mice received gentamycin (0.8 mg/ml in drinking water) starting 10 days before irradiation and maintained during 2 weeks. 8 weeks after bone marrow transplantation, mice were challenged with 3-MCA.

#### Sarcoma transplantable models

FS6 and MN-MCA1 cell lines were cultured in RPMI-1640 medium supplemented with 10% Fetal Bovine Serum (FBS) 1% L-Glutammine, 1% Pen/Strept. On the day of the experiment cells were detached with Tripsin/EDTA solution (Lonza, Basel, Switzerland), washed twice in PBS^−/−^ and diluted in PBS^−/−^ before injection. Mice were anesthetized and shaved on the back, 2x10^6^ FS6 or 5x10^5^ MN-MCA1 were injected subcutaneously. In the *in vivo* cotransfer experiments cells were co-injected with FACS-sorted 1x10^5^ DNT_αβ_ cells isolated from spleen of healthy *Csf3r*^*+/+*^ mice.

#### Undifferentiated pleomorphic sarcoma patients

Patients (n = 19) whose biological samples were included in the study gave their signed consent to donate the tissue remaining after the diagnostic procedure. Human UPS surgical samples were collected from Humanitas Biobank (n = 19), upon approval by the Humanitas Research Hospital Ethical Committee (authorization 609/17, issued on 18 December 2017). Informed consent was obtained from all participants. Demographics and clinicopathologic features of recruited patients are described in Table S7.

### Method Details

#### Organ collection, digestion and flow cytometry analysis

Single-cell suspensions of sarcomas, blood, spleen and skin were obtained and stained. Sarcomas were manually disaggregated and incubated with 0.1 mg/mL Type IV Collagenase (Sigma Aldrich) in PBS^−/−^ at 37°C for 60 minutes. Cell suspensions from spleen were obtained as previously described (Ponzetta et al., 2015). Dorsal skin samples from untreated mice and 3-MCA injection sites were manually disaggregated and incubated with 0.35 mg/mL DNase (Roche, Basel, Switzerland) and 0.125 mg/mL Liberase TM (Roche) in RPMI at 37°C for 90 minutes. Before further procedures, cell suspensions from every organ were filtered using 70 μm cell strainers (Corning, Corning, US). Extracellular staining was performed using a PBS^−/−^ buffer containing FCS 2%, EDTA 2mM and NaN_3_ 0.05%. Prior to any surface staining, cells were incubated with Aqua LIVE/Dead-405 nm staining (Invitrogen, Carlsbad, US) or Live/Dead Fixable Dye eF780 (ThermoFisher, Waltham, US) and negative cells were considered viable. Then Fc blocking reagent (Clone 24G2) was added to any cell suspension for 10 minutes at 4°C. Finally, extracellular staining was performed, and antibody mix was added to cell suspension for 20 minutes at 4°C. The following murine antibodies were used: anti-CD45-BV605, -BV650 or -PerCp-Cy5.5 (Clone 30-F11); anti-CD3e-PerCP-Cy5.5 or -APC (Clone 145-2C11); anti-CD45.2-BUV805 (Clone 104); anti-CD19-PE, - PerCP-Cy5.5 or -eFluor450 (Clone 1D3); anti-NK1.1-APC, -PE, -PECF594 or -BV650 (Clone PK136); anti-CD11b-BV421, -BV480, -BV786, -APCCy7, -FITC (Clone M1/70); anti-CD27-FITC, –APC-eFluor780 or PE-Cy7 (Clone LG.7F9); anti-CD4-AlexaFluor700 (Clone RM 4-5) or -FITC (Clone H129.19); anti-CD8-PE, BV480 or -BV570 (Clone 53-6.7); anti-iFNγ-Alexa700 or -BV421 (Clone XMG1.2), anti-Ly6G-PECF594, -BUV395 (Clone 1A8), anti-Ly6C-FITC or –BV421 (Clone AL-21), anti-MHCII-PercP-Cy5.5, -BV711 (Clone 2G9) or –FITC (Clone M5/114.15.2), anti-F4/80-PECy7 (Clone BM8), anti-CD103-PercP-Cy5.5 (Clone 2E7), CD86-eF450 (Clone GL-1), anti-CD24-APCeF780 (CloneM1/69), anti-CD64-PE (Clone X54-5/7.1), anti-CD11c-AlexaFluor700 or -PE (Clone HL3), anti-TCRβ-BV711 (Clone H57-597), anti-TCRγδ-BV421 or –PercP-Cy5.5 (Clone GL3), anti-T-bet-PE or –BV785 (Clone O4-46), anti-Eomes-AlexaFluor488 (Clone Dan11Mag), anti-Rorγt-PECF594 (Clone Q31-378), anti-PLZF-AlexaFluor674 (Clone R17-809), anti-CD54-PE (Clone 3E2), anti-CD49a-BV711 (Clone Ha31/8), anti CD49b-APC (CloneDX5), anti-KLRG1-BV786 (Clone 2F1), anti-NKP46-BV421 (Clone 29A1.4), anti-Ly49F-BV421 (Clone HBF-719); anti-Ly49C-I-BV605 (Clone 5E6); anti-Ly49A-BUV395 (Clone A1); anti-Ly49G2-BV480 (4D11), anti-NKG2A/C/E-BUV563 (Clone20d5); anti-CD94-BV650 (Clone 18d3); anti-CD62L-APC or –BV570 (MEL-14) from BD Bioscience (San Jose, US), ThermoFisher (Waltham, US), BioLegend (San Diego, US) or Miltenyi Biotec (Bergisch Gladbach, Germany). Murine iNKT cells were detected using CD1d-APC tetramers loaded with αGalCer (ProImmune, Oxford, UK), while murine MAIT cells were detected using MR1-BV421 or -PE tetramers loaded with 5-OP-RU (kindly provided by James McCluskey, University of Melbourne). To avoid non-specific tetramer binding, during MAIT staining cells were pre-incubated with the irrelevant unconjugated tetramer MR1 loaded with 6-FP (as described in Corbett et al., 2014, Rahimpour et al., 2015, Reantragoon et al., 2013). Foxp3/Transcription Factor Staining Buffer Set (ThermoFisher) was used for intracellular staining of transcription factors and cytokines. Results are reported as frequency or as mean fluorescence intensity (MFI) normalized on isotype control or fluorescence minus one (FMO). Cells were analyzed on LSR Fortessa (BD Bioscience), for some experiments cells were analyzed on FACSymphony (BD Bioscience). Data were analyzed with FlowJo software (Treestar, Ashland, US).

#### PrimeFlow RNA Assay

PrimeFlow RNA Assay (ThermoFisher Scientific) was performed following manufacturer’s instructions. Briefly, a single-cell suspension was obtained from dorsal skin of 3-MCA-treated mice and stained for surface antigens as described above. After fixation and permeabilization, *il12a*-AlexaFluor647-, *il12b*-AlexaFluor488- or *actb*-AlexaFluor647-conjugated target probes were added for hybridization, that was performed at 40°C for 2 hours. Signal amplification was achieved by adding PrimeFlow RNA Pre-Amp mix for 1.5 hours at 40°C and then PrimeFlow RNA Amp Mix for 1.5 hours at 40°C.

#### *In vivo* EdU-based proliferation assay

EdU (5-ethynyl-2′-deoxyuridine) was administered i.p. (0.5mg/mice) to 3-MCA-treated mice, 24 hours before sacrifice once they reached the experimental endpoint (calculated tumor volume of 2000 mm^3^). A single-cell suspension was obtained and surface antigen staining was performed. EdU staining was performed with Click-iT EdU AlexaFluor647 Flow Cytometry Assay Kit (ThermoFisher Scientific) following manufacturer’s instructions.

#### Bone-marrow derived macrophages (BMDMs)

Mouse bone-marrow cells were isolated from femurs and tibiae of *Csf3r*^*+/+*^ mice and cultured at density of 1.5x10^6^ cells/mL in RPMI-1640 medium supplemented with 10% Fetal Bovine Serum (FBS) 1% L-Glutammine, 1% Pen/Strept, with 20 ng/mL of murine GM-CSF or 25 ng/mL of murine M-CSF (both M-CSF and GM-CSF were purchased from Peprotech). Cells were washed with PBS and medium replaced at day 2 and 5. BMDMs were stimulated on day 7 with murine IFNγ (20 μg/mL) or murine IL-4 (20 μg/mL), alone or in combination with murine G-CSF (50 ng/mL). After 24 hours cells were lysed with Trizol for further mRNA quantification.

For co-culture experiments, GM-CSF-derived BMDMs were stimulated with GM-CSF (50 ng/mL), either alone or in combination with CpG (250 nM) (Invivogen, San Diego, US) or STING agonist cAIMP (1 μg/mL) (Invivogen). In indicated conditions, BMDMs were co-cultured with 3x10^5^ FACS-sorted neutrophils for 24 hours. In transwell experiments neutrophils were added into the upper compartment of a Transwell permeable support with 0.4 μm pore (Corning, NY, US). After 24 hours medium was collected and IL-12p70 concentration was analyzed by ELISA.

#### *Ex vivo* functional assays

Tumor-infiltrating leukocytes were MACS-enriched using CD45 Microbeads (Miltenyi Biotec) according to manufacturer’s instructions, obtaining a purity ≥ 85%. Enriched cells were cultured in RPMI-1640 medium supplemented with 10% Fetal Bovine Serum (FBS) 1% L-Glutammine, 1% Pen/Strept and IFNγ intracellular staining was performed upon 5 hours of treatment with Cell Stimulation Cocktail 1X (ThermoFisher), using Foxp3/Transcription Factor Staining Buffer Set (ThermoFisher). BD GolgiPlugTM (containing Brefeldin) was added 4 hours prior to intracellular staining.

Cytokines stimulation experiments were performed on MACS-enriched CD45^+^ Tumor-infiltrating leukocytes, that were previously depleted of CD11b^+^ cells (CD11b Microbeads, Miltenyi Biotec). Enriched cells were stimulated with the indicated combinations of IL-2 (Proleukin, Novartis) (10ng/mL), recombinant murine IL-12 (Peprotech, London, UK) (20 ng/mL), human IL-18 (MBL, Woburn, US) (50 ng/mL) for 16 hours. BD GolgiPlugTM was added during the last 4 hours of stimulation. Intracellular staining was performed as indicated before using Foxp3/Transcription Factor Staining Buffer Set (ThermoFisher).

Purified splenic T cells from healthy *Csf3r*^+/+^ mice at a concentration of 3x10^6^ cells/mL were stimulated with recombinant murine IL-12 (Peprotech, London, UK) (20 ng/mL) and/or human IL-18 (MBL, Woburn, US) (50 ng/mL) for 16 hours. BD GolgiPlugTM (containing Brefeldin) was added during the last 4 hours of stimulation.

In a second set of experiment, purified splenic T cells from healthy *Csf3r*^+/+^ mice were cultured with conditioned medium (CM) from BMDMs and neutrophils co-cultures at a concentration of 3x10^6^ T cells/mL. Rat anti-iL-12p75 (Clone R2-9A5; Rat Isotype Control, Clone LTF-2) 20 μg/mL were added to the CM and incubated at 4°C for 30 minutes, before the incubation with T cells. Cells were incubated for 24 hours, and BD GolgiPlugTM was added during the last 4 hours of stimulation.

#### RNA purification

For qPCR experiments, total RNA was extracted using Trizol reagent (Invitrogen) following the manufacturer’s recommendations. RNA was further purified using RNeasy Min-elute RNA isolation kit (QIAGEN, Hilden, Germany). For 3′ mRNA sequencing experiments, RNA was purified with Maxwell 16 LEV SimplyRNA Cells Kit (Promega, Madison, US) using Maxwell 16 Instrument (Promega).

#### Quantitative PCR

cDNA was synthesized using 2 μg of total RNA by reverse transcription using High Capacity cDNA archive kit (Applied Biosystems, Foster City, US) and quantitative real-time PCR was performed using the SybrGreen PCR Master Mix (Applied Biosystems) in a CFX96 TouchTM Real-Time PCR Detection System (Bio-Rad, Hercules, US). Data were analyzed with the Δ^2^CT method (Applied Biosystems, Real-Time PCR Applications Guide). Data were normalized based on the GAPDH expression determined in the same sample. Analysis of all samples was performed in duplicate. Primers were designed according to the published sequences and listed as follows:

*Retnla* (*Fizz1*): forward 5′-CCC TTC TCA TCT GCA TCT CC-3′, reverse 5′-CTG GAT TGG CAA GAA GTT CC-3′; *Chil3* (*Ym1*): forward 5′-TCT GGG TAC AAG ATC CCT GAA-3′, reverse 5′-TTT CTC CAG TGT AGC CAT CCT T-3′; *Ccl5*: forward 5′-TGC AGA GGA CTC TGA GAC AGC-3′, reverse 5′-GAG TGG TGT CCG AGC CAT A-3′; *Nos2*: forward 5′-GCC ACC AAC AAT GGC AAT A-3′, reverse 5′-CGT ACC GGA TGA GCT GTG AAT T-3′; *Ccl2*: forward 5′- ATT GGG ATC ATC TTG CTG GT-3′, reverse 5′- CCT GCT GTT CAC AGT TGC C-3′; *Arg1*: forward 5′- TTT TTC CAG CAG ACC AGC TT-3′, reverse 5′- AGA GAT TAT CGG AGC GCC TT-3′; *Tgfb1*: forward 5′-CAA CCC AGG TCC TTC CTA AA-3′, reverse 5′- GGA GAG CCC TGG ATA CCA AC-3′; *Il27p28*: forward 5′- AGC TCT TGA AGG CTC AGG G-3′, reverse 5′- GTG ACA GGA GAC CTT GGC TG-3′; *Ifng*: forward 5′-TCA AGT GGC ATA GAT GTG GAA GAA-3′, reverse 5′-TGG CTC TGC AGG ATT TTC ATG-3′; *Il10*: forward 5′-GGT TGC CAA GCC TTA TCG GA-3′, reverse 5′-ACC TGC TCC ACT GCC TTG CT-3′; *Stab1*: forward 5′-CCC TCC TTC TGC TCT GTG TC-3′, reverse 5′- CAA ACT TGG TGT GGA TGT CG-3′; *Mrc1*: forward 5′-TGG CAT GTC CTG GAA TGA T-3′, reverse 5′-CAG GTG TGG GCT CAG GTA GT-3′; *Msr1*: forward 5′-TGC CCT CAT TGC TCT CTA CC-3′, reverse 5′- TTC ATT TCC CAA TTC AAA AGC TC-3′; *Il4ra*: forward 5′- GTG GAG CCT GAA CTC GCA-3′, reverse 5′-AAG CAC GCA GAT CCA AAA TC-3′; *Marco*: forward 5′-TTC TGT CGC ATG CTC GGT TA-3′, reverse 5′-CAG ATG TTC CCA GAG CCA CC-3′; *Met*: forward 5′-TGT CCG ATA CTC GTC ACT GC-3′, reverse 5′-CAT TTT TAC GGA CCC AAC CA-3′; *Il1b*: forward 5′- GGT CAA AGG TTT GGA AGC AG-3′, reverse 5′- TGT GAA ATG CCA CCT TTT GA-3′; *Ccl3*: forward 5′- GTGGAATCTTCCGGCTGTAG-3′, reverse 5′- ACCATGACACTCTGCAACCA-3′; *Cxcl10*: forward 5′-CCG TCA TTT TCT CCC TCA TCC-3′, reverse 5′- CCC TAT GGC TGC TTC ACT CTC A-3′; *Il23a*: forward 5′-AGC ATT TAT GCT TCC AAA GC-3′, reverse 5′-GGA GGT CTC AAG TTC CTA CAT G-3′; *Ccr3*: forward 5′-TGA AAC TGT GAT CTT GGG ACA-3′, reverse 5′-CAG CAT CAA CAA CAC GTT CC-3′; *Il1r5*: forward 5′-GAT GCA TGT TTA GGC TTC CA-3′, reverse 5′-TCT TCT GCT GTC TGG AGC AA-3′; *Il1r7*: forward 5′-AGA GTG CAG AGA GGC AAA CC-3′, reverse 5′-TGA AAC CCT CTT CCT CCA GA-3′; *Il12rb1*: forward 5′- GCA GCC GAG TGA TGT ACA AG-3′, reverse 5′- GAG ACG CGA AAA TGA TGG AT-3′; *Il12rb2*: forward 5′- AAC ACC AGA GGA AGA GCC TG-3′, reverse 5′- CGT CAC CTG ATA GTG GAG GA-3′; *Gapdh/GAPDH*: forward 5′-GCA AAG TGG AGA TTG TTG CCA T-3′, reverse 5′-CCT TGA CTG TGC CGT TGA ATT T-3′.

#### 3′-mRNA Sequencing

Tumor-infiltrating CD4^+^, CD8^+^, UTC_αβ_ and γδ T cells from *Csf3r*^+/+^ and *Csf3r*^−/−^ sarcomas were FACS sorted (quadruplicates for *Csf3r*^−/−^ and for *Csf3r*^−/−^ mice), and RNA was prepared as described above.

Total RNA extracted from purified T cell subsets were subjected to Poly(A) mRNA sequencing. Libraries were constructed using the SMART Seq v4 Ultra Low Input RNA kit according to manufacturer's instruction (Illumina, San Diego, US). Sequencing was performed with the NextSeq 500 (Illumina). All libraries were sequenced in single-end mode (75bp length).

#### Single-cell RNA sequencing

UTC_αβ_ sorted populations in *Csf3r*^−/−^ and *Csf3r*^+/+^ mice were subjected to single-cell RNA sequencing analysis. Two biological replicates for each condition (*Csf3r*^*−/−*^ and *Csf3r*^*+/+*^) were analyzed. Single cell suspensions of cells have been prepared by tissue mincing and enzyme digestion. FACS-sorted UTC_αβ_ were counted with an automatic cell counter (Countess II, Thermo Fisher). UTC_αβ_ were loaded into one channel of the Single Cell Chip A using the Single Cell 3′ reagent kit v2 (10X Genomics) for gel bead emulsion generation. Following capture and lysis, cDNA was synthesized and amplified for 14 cycles following the manufacturer’s protocol (10X Genomics). 50 ng of the amplified cDNA were then used for each sample to construct Illumina sequencing libraries. Sequencing was performed on the NextSeq500 Illumina sequencing platform following 10x Genomics instruction for reads generation. We recovered a total number of 15,137 cells from *Csf3r*^*+/+*^ and 17,388 cells from *Csf3r*^*−/−*^ samples. An average sequencing depth of 20,000 reads/cell was recovered for each sample we processed. Cells with a null *Cd3e* unique molecular identifier (UMI) count were not considered further for the analysis, resulting in a total number of 31,623 cells (14,721 cells for *Csf3r*^*+/+*^ and 16,902 cells for *Csf3r*^*−/−*^ samples).

#### Purification of murine leukocytes

Bone marrow neutrophils were MACS-enriched with Neutrophil Isolation Kit (Miltenyi Biotec) according to manufacturer’s instructions for adoptive transfer experiments. Purity of neutrophils was > 98% as determined by flow cytometry.

For *in vitro* co-cultures experiments MACS-enriched neutrophils were stained for LiveDead-Fixable Dye eF780; anti-CD45-BV605; anti-CD11b-BV421; anti-Ly6G-PECF594 and sorted on a FACSAria cell sorter (BD Bioscience) to obtain a highly purified neutrophils population (purity > 99%).

Splenic T cells were MACS enriched with Pan T cell Isolation kit II (Miltenyi Biotec) according to manifacturer’s instructions, for *ex vivo* stimulation with cytokines. Purity of the obtained CD3+ cells was > 99%. In some experiments, splenic T cells were MACS enriched and subsequently T cell subsets (CD4^+^, CD8^+^, UTC_αβ_ and γδ T cells) were FACS-sorted. Resulting cells were processed for mRNA extraction.

For RNA expression analysis on neutrophils, macrophages, immature macrophages and monocytes, tumor-associated CD11b^+^ cells were MACS-enriched (CD11b Microbeads, Miltenyi Biotec) according to manufacturer’s instructions. CD11b+ cells represented more than 85% of resulting cell suspension. Next, neutrophils, macrophages, immature macrophages and monocytes were stained with LiveDead-Fixable Aqua, anti-CD45-BV605, anti-CD11b-BV786, anti-Ly6G-PECF594, anti-Ly6C-FITC, anti-F4/80-PECy7, anti-CD64-PE, anti-CD11c-AlexaFluor700 and sorted on a FACSAria cell sorter (BD Bioscience) to obtain high purity myeloid populations. Purity of each population was > 98%. Resulting cells were processed for mRNA extraction.

For RNaseq experiments on T cell subset populations, tumor cells were depleted of CD11b^+^ cells and resulting cells were stained with LiveDead-Fixable Aqua, anti-CD45-BV605, anti-CD11b-APCCy7, anti-TCRβ-BV711, anti-TCRγδ-BV421, anti-CD4-AlexaFluor700 and anti-CD8-BV570 and sorted on a FACSAria cell sorter to obtain high purity T cell subset populations. Purity of each population was > 98%. Resulting cells were processed for mRNA extraction.

For single cell RNaseq experiments on UTC_αβ_, tumor infiltrating leucocytes from *Csf3r*^+/+^ and *Csf3r*^−/−^ mice were MACS-enriched (CD45^+^ TIL Microbeads), then CD11b^+^ cells and CD4^+^/CD8^+^ T cells were depleted. Cells were FACS-sorted as CD45^+^/CD11b^-^/TCRβ^+^/TCRγδ^-^/CD4^-^/CD8^-^ cells. Resulting cells (purity < 99.9%) were further processed for single cell RNaseq.

For the *in vivo* cotransfer experiments, splenic T cells were first MACS-enriched with Pan T cells Isolation kit II; after negative selection of CD4^-^/CD8^-^ T cells (CD4^+^/CD8^+^ T cells Microbeads, Miltenyi Biotec) cells were stained with fluorophore-conjugated antibodies and DNT _αβ_ cells were FACS-sorted on a FACSAria as CD45^+^/CD11b^-^/CD19^-^/TCRβ^+^/TCRγδ^-^/CD4^-^/CD8^-^/MR1-OP-RU^-^/CD1d-αGal/Cer^-^ cells. Cell purity was < 99.0%.

#### Cytokine Measurement

Tumors or 3-MCA injection sites were homogenized in 1 mL PBS^−/−^ containing protease inhibitors (Complete-EDTA-free; Roche) and PMSF (1mM). Tissue homogenates were centrifuged at 14000 rpm for 30 min at 4°C and supernatants were stored at −20°C for cytokine analysis. Murine IFNγ, CCL2, CXCL1, CXCL2, CXCL12, M-CSF, G-CSF, TNFα, IL-22, IL-1β, IL-6, IL-12p70, VEGF, TGFβ and IL-23p19 were measured in tissue homogenates by ELISA (R&D DuoSet ELISA Development System) according to manufacturer’s instructions. Murine IL-18, IL-17A were measured in tissue homogenates by ProcartaPlex Assay (ThermoFisher) according to manufacturer’s instructions.

#### Immunohistochemistry

PFA-fixed, paraffin-embedded mouse tumor tissues were analyzed for each condition. Consecutive sections from the middle of the tissue were used for histological examination in each mouse. Paraffin-embedded tissue sections were mounted on Super-frost slides, dewaxed in xylene and rehydrated in ethanol. Endogenous peroxidase was blocked for 20 min in 90% ethanol containing 2% H_2_O_2_. Sections were then pretreated in a microwave oven (two cycles for 3 minutes each at 800 W, in 0.25 mM EDTA buffer). Unspecific sites were blocked with Rodent Block M (Biocare Medical) 30 minutes and tissues were incubated for two hours with affinity-purified Ig against CD31 (Clone MEC13.3; BD Bioscience) in PBS supplemented with BSA (1%) and NP40 (0.02%). Envision+ System HRP Labeled Polymer anti Rabbit (Dako), Rat on Mouse HRP-Polymer kit (Biocare Medical) was used as secondary antibody. For anti-human CD66b staining, paraffin embedded tissues were cut at 3 μm and put over night at 37°C. Then sections were dewaxed in xylene and rehydrated in ethanol. Antigen unmasking was performed in Decloaker Chamber in DIVA Buffer (Biocare Medical) (3 minutes at 125°C, 5 minutes at 90°C). Endogenous peroxidase was blocked with H_2_O_2_ 2% for 20 minutes. Unspecific binding sites were blocked with Background Sniper (Biocare Medical) for 15 minutes. Mouse anti-human CD66b (Clone G10F5; BD PharMingen, CA) or control antibody was diluted in Washing Buffer (PBS pH 7.00 + 0.05% Tween 20) and incubated in humid chamber 1h. MACH 1 HRP polymer (Biocare Medical) was used as secondary antibody. After washing, slides were developed with DAB (3,3′-diaminobenzidine) (Biocare Medical) and counterstained with Hematoxilyn. Tissues were dehydrated with ethanol, mounted with Eukitt and analyzed with an Olympus BX61 virtual slide scanning system. The number of CD66b^+^ cells infiltrating the tumors was determined in whole tissue sections (magnification 20X) and mean values were employed to divide tumors in CD66b^high^ and CD66b^low^.

### Quantification and statistical analysis

#### Statistical analysis

For animal studies, sample size was defined on the basis of past experience on cancer models, to detect differences of 20% or greater between the groups (10% significance level and 80% power). Values were expressed as mean ± s.e.m. Wilcoxon matched-pairs signed-rank test was used to compare two groups for tumor incidence. Friedman test with Dunn’s multiple comparison was used to compare tumor incidence for multiple group experiments. One-way ANOVA or Kruskal-Wallis test were used to compare multiple groups. Two-tailed multiple Student’s t test was used to compare unmatched groups with Gaussian distribution. Two-tailed Mann–Whitney *U*-test was used to compare unmatched groups with non-Gaussian distribution. The Kaplan-Meier method was used for survival curve analysis, and the log-rank (Mantel-Cox) test was used to determine the statistical significance. p ≤ 0.05 was considered significant. A ROUT test was applied to exclude outliers. Statistics were calculated with GraphPad Prism version 7, GraphPad Software.

#### 3′-mRNA Sequencing analysis

Raw reads were preprocessed for adaptor trimming and quality check was assessed using the FastQC tool (http://www.bioinformatics.babraham.ac.uk/projects/fastqc). Reads were aligned to the reference genome (Ensembl Mouse release GRC38) using the STAR (Dobin et al., 2013) algorithm. To minimize the variability among replicates, a coefficient of variation (CV) was calculated based on TMM (Trimmed Mean of M-Values) normalized expression values in each group. Genes exceeding 100% CV in at least one replicate’s group were not considered for further differential expression analysis. Differential expression analysis was performed using the GLM approach implemented in the R/Bionconductor (Gentleman et al., 2004) edgeR (Robinson et al., 2010) package (*p value* ≤ 0.001). Results of differential analysis are provided in Table S2. The resulting gene lists were analyzed through the use of Ingenuity Pathways Analysis (IPA) (Krämer et al., 2014). Results of IPA analysis are provided in Table S3.

#### Single cell RNA sequencing analysis

Raw sequencing data in the format of bcl files were converted in fastq files and aligned to the mouse reference genome [http://cf.10xgenomics.com/supp/cell-exp/refdata-cellranger-mm10-1.2.0.tar.gz] taking advantage of the Cell Ranger Pipeline version 2.1, provided by 10X Genomics. Raw digital gene expression matrix (unique molecular identifier (UMI) counts per gene per cell) were analyzed using the Seurat R package version 2.3.0 with default parameters. To assess the correlation between biological replicates in each condition we computed a Pearson correlation analysis using normalized gene expression matrices (Figure S5L-M). Based on high correlation between replicates, samples belonging to each condition were pooled together using the Cell Ranger aggregate option producing a unique raw digital gene expression matrix in *Csf3r*^*−/−*^ and *Csf3r*^*+/+*^ conditions. Pooled data were imported into the Seurat pipeline and filtered applying the following thresholds: less than 200 or more than 3500 as unique expressed genes, more than 20000 as the number of UMIs and 10% as the percentage of mitochondrial genome content (for *Csf3r*^*+/+*^ condition) and less than 200 or more than 4000 as unique expressed genes, more than 20000 as the number of UMIs and 10% as the percentage of mitochondrial genome content (for *Csf3r*^*−/−*^ condition). Cell cycle phases were predicted and corrected in each dataset using a Seurat function that scores each cell based on the expression of canonical marker genes for S and G2/M phases. The resulting dataset was normalized through a global-scaling method, converted by a scale factor (10,000 by default) and log-transformed using the ScaleData Seurat implemented function. A canonical correlation analysis was run in order to identify common sources of variation between the two datasets, using the RunCCA implemented function that stores into a single object the canonical correlation vectors. Finally an integrated analysis of the two datasets was performed choosing a number of 30 correlation components. The resulting data were subjected to clustering analysis using standard Seurat package procedures. A validation consistency procedure of the resolution (using the ValidateCluster function) allowed us to select 0.6 as the suitable resolution level. The total 12 identified clusters were visualized using t-distributed Stochastic Neighbor Embedding of the principal components (t-SNE) as implemented in Seurat.

Average gene expression matrices were retrieved for each cluster and a Spearman correlation analysis was performed, using the Picante R package version 1.6-2. Differential expression analysis among clusters (using the FindAllMarkers implemented function, with parameters only.pos = FALSE, min.pct = 0.2, thresh.use = 0.2) allowed us to select the top markers expressed at a higher level by each cluster.

Pathway enrichment analysis examining enriched processes in clusters was performed using Gene Set Variation Analysis (GSVA) software (Hänzelmann et al., 2013) from Bioconductor (version 3.8). Mouse_AllPathways_November_01_2018_symbol.gmt from [http://download.baderlab.org/EM_Genesets/current_release/] was used to identify enriched cellular pathways in GSVA.

#### Single-cell trajectory analysis

Monocle (Qiu et al., 2017, Trapnell et al., 2014) was used to investigate transcriptional and functional trajectories concerning the twelve clusters identified with Seurat (see Cell clustering, differential expression and pathway analysis paragraph). Using the reversed graph embedding approach, Monocle learns the kinetics of gene expression and places each cell along an inferred trajectory. The data, in the format of raw data, together with clusters information deriving from Seurat, were loaded into a Monocle object. Normalization and dimensionality reduction were performed using default parameters. The trajectory was designed using the plot_cell_trajectory command. Cells were ordered along an artificial trajectory based on gene expression changes among clusters. Genes that underwent a significant change along pseudotime (q-value < 0.05) were visualized by a heatmap using plot_pseudotime_heatmap. The trend of *Klra1*, *Klra6*, *Klra7* and *Klrd1* gene expression was depicted by individual graphs using the plot_genes_in_pseudotime function.

#### Public gene expression data analysis

Publicly available RNA sequencing data were downloaded from the Cancer Genome Atlas database (TCGA) through the Firebrowse repository (http://firebrowse.org/; release 01/28/2016), for Sarcoma dataset (SARC) and from Xena Browser (https://xenabrowser.net/) for Melanoma dataset (SKCM).

The sarcoma dataset included a total of 261 tumor samples, classified in the following subtypes: dedifferentiated liposarcoma (n = 58), desmoid tumor (n = 2), giant cell sarcoma (n = 1), leiomyosarcoma (lms, n = 104), malignant peripheral nerve sarcoma (mpnst, n = 10), myxofibrosarcoma (n = 25), synovial sarcoma (n = 10), undifferentiated sarcoma (ups, n = 51). Sarcoma histotypes with a total number of samples < 10 were excluded from further analysis. The Melanoma dataset included a total of 474 patients. RSEM (RNASeq by Expectation-Maximization) expression values were retrieved and log-transformed. For colorectal cancer (CRC) cohort we used a microarray dataset (GEO: GSE24551) including a total of 160 samples. For ovarian cancer we used a microarray dataset (GEO: GSE32062) including a total of 270 samples, from which we selected the high grade cohort (128 samples). For both datasets we used respectively Robust Multi-array Average (RMA) and Normalized Signal Intensity (NSI) gene expression values as provided by GEO.

Survival analysis was performed with GraphPad Prism™ using the Kaplan-Meier (KM) approach and applying the Log-rank (Mantel-Cox) test to estimate survival curves comparison. For *CSF3R* survival analysis, the median gene expression value was used to classify tumor samples into *CSF3R*^low^ and *CSF3R*^high^ gene expression groups. A signature of 31 neutrophil-related genes was retrieved from Bindea et al. (Bindea et al., 2013) (Table S6). This neutrophil-specific gene signature was based on previous microarray datasets of gene expression in resting and activated human peripheral blood leukocyte subsets (Chtanova et al., 2005). In particular, the signature is composed of genes with higher expression in resting and LPS-activated neutrophils compared to any other leukocyte subset. Of note, *CSF3R* was included in the neutrophil signature and, in the TCGA dataset of UPS patients, a significant correlation was observed between *CSF3R* expression and 28 out of 30 genes present in the neutrophil signature (not shown). Type 1 immune response gene signature was designed by including factors known to be upstream or downstream of *IFNG* signaling pathway (genes listed in Table S6) (Ayers et al., 2017, Ikeda et al., 2002, Murphy et al., 2000, Swain et al., 2012). Their expression correlated with *IFNG* (p < 0.005, not shown). For both signatures, RSEM or normalized signal values were converted into z-score applying the following transformation: z-score = (X – average(X))/stdev(X), where X represents gene expression values. The average and standard deviation were calculated considering the expression of X across all tumor samples, as previously described (Chao et al., 2016). Finally, the median z-score value was considered as the threshold to define Low and High expression groups.

#### Statistics and reproducibility

Figures 1A and 1B, n = *10* mice per group; Figure 1C, n = 20 (*Csf3r*^+/+^), n = 8 (*Csf3r*^−/−^), n = 12 (*Csf3r*^−/−^ + Nϕ). Figures 1A and 1B, one representative experiments out of twenty performed; Figure 1C, pooled data of two independent experiments performed.

Figure 2A, n = 9 mice per group; Figures 2B and 2C, n = 8 (*Csf3r*^+/+^ isotype), n = 9 (*Csf3r*^+/+^ anti-CD115), n = 9 (*Csf3r*^−/−^ isotype) or n = 10 (*Csf3r*^−/−^ anti-CD115) mice per group, Figure 2D, n = 5 (*Csf3r*^+/+^), n = 5 (*Csf3r*^−/−^), n = 4 (*Csf3r*^−/−^ + Nϕ); Figure 2E, n = 10 (*Csf3r*^+/+^), n = 10 (*Csf3r*^−/−^), n = 4 (*Csf3r*^−/−^ + Nϕ) Figure 2F, n = 9 mice per group; Figures 2A–2F, one experiment performed.

Figure 3A, n = 12 (*Csf3r*^+/+^) or n = 18 (*Csf3r*^−/−^) mice; Figure 3C, n = 16 (*Csf3r*^+/+^) or n = 21 (*Csf3r*^−/−^) mice; Figure 3D, n = 5 (Csf3r^+/+^), n = 14 (*Csf3r*^−/−^), n = 5 (*Csf3r*^−/−^+neutrophils); Figure 3E, n = 11 (*Csf3r*^*+/+*^), n = 14 (*Csf3r*^−/−^), n = 14 (*Csf3r*^−/−^ + neutrophils) mice; Figure 3F, n = 9 (*Csf3r*^+/+^), n = 20 (*Csf3r*^−/−^) mice per group, Figure 3G, n = 5 (*Csf3r*^+/+^), n = 14 (*Csf3r*^−/−^), n = 5 (*Csf3r*^−/−^ + NΦ) mice per group. Pooled data of two (Figures 3A, 3C, 3E, and 3F) experiments are shown. Figures 3D and 3G, one experiment performed.

Figure 4A, n = 4 mice per group; Figures 4B and 4C, n = 4 mice per group; Figure 4D, n = 3 mice per group; Figure 4E, n = 6 (*Csf3r*^+/+^ isotype), n = 7 (*Csf3r*^+/+^ anti-iL-12p70), n = 5 (*Csf3r*^−/−^ isotype) or n = 4 (*Csf3r*^−/−^ anti-iL-12p70) mice per group; Figure 4G, n = 5 (Mϕ), n = 5 (Nϕ), n = 8 (Mϕ+Nϕ), n = 3 (Mϕ+Nϕ Transwell); Figure 4H, n = 4 mice per group; Figure 4I, n = 10 mice per group. Figures 4A–4F, one experiment performed. Pooled data of two (Figures 4G and 4H) or three (Figure 4I) experiments are shown.

Figures 5A–5E, n = 2 mice per group; Figures 5F and 5G, n = 5 mice per group. Figures 5A–5E, one experiment performed.

Figures 6A–6D, n = 2 mice per group; Figures 6E and 6F, n = 5 mice per group; Figure 6G, n = 8 (*Csf3r*^+/+^), n = 7 (*Csf3r*^+/+^ + DNT_αβ_); Figure 6H, n = 8 (*Csf3r*^+/+^), n = 7 (*Csf3r*^+/+^ + DNT_αβ_). Figures 6A–6H, one experiment performed.

### Data and Software Availability

#### Data availability and accession

The accession number for the bulk RNA-sequencing data reported in this paper is Gene Expression Omnibus (GEO) (https://www.ncbi.nlm.nih.gov/geo/): GSE109031. The accession number for the single cell RNA-sequencing data reported in this paper is Gene Expression Omnibus (GEO): GSE123508.

The authors declare that all other data supporting the findings of this study are available within the article and its supplementary information files.

#### Reagent availability

Reagents used in the present study are described in the Key Resources Table.

## Supplemental Information

Document S1. Tables S1, S6, and S7

Table S2. Differentially Expressed Genes between Sorted Sarcoma-Infiltrating *Csf3r*^−/−^ and *Csf3r*^+/+^ UTC_αβ_ and Differentially Expressed Genes between sorted Sarcoma-Infiltrating *Csf3r*^+/+^ UTC_αβ_ and CD4^+^, CD8^+^, and γδ T Cells, Related to Figure 4

Table S3. IPA Analysis of Significantly Enriched Biological Pathways and Prediction of Upstream Regulators in *Csf3r*^−/−^ and *Csf3r*^+/+^ Sarcoma-Infiltrating T Cell Subsets, Related to Figure 4

Table S4. List of Differentially Expressed Genes in Clusters 1–12, Related to Figure 5 Genes were filtered for Average Log Fold Change (AvgLFC) > 0 and for Adjusted p value < 0.05. Genes are ranked according to their AvgLFC.

Table S5. List of Differentially Expressed Genes along Cell Trajectory in Monocle, Related to Figure 6 Genes are divided in six gene sets according to their common expression trend along the pseudotime.
