# Supplementary material for: Neutrophils Driving Unconventional T Cells Mediate Resistance against Murine Sarcomas and Selected Human Tumors
Source: Cell. 2019 Jul 11;178(2):346–360.e24. doi: 10.1016/j.cell.2019.05.047 (PMC6630709; doi:10.1016/j.cell.2019.05.047)
Supplement: Document S1. Tables S1, S6, and S7 [file mmc1.pdf]

Cell, Volume *178*

Supplemental Information

Neutrophils Driving Unconventional T Cells Mediate Resistance against Murine Sarcomas and Selected Human Tumors

Andrea Ponzetta, Roberta Carriero, Silvia Carnevale, Marialuisa Barbagallo, Martina Molgora, Chiara Perucchini, Elena Magrini, Francesca Gianni, Paolo Kunderfranco, Nadia Polentarutti, Fabio Pasqualini, Sabrina Di Marco, Domenico Supino, Clelia Peano, Ferdinando Cananzi, Piergiuseppe Colombo, Silvana Pilotti, Suliman Yousef Alomar, Eduardo Bonavita, Maria Rosaria Galdiero, Cecilia Garlanda, Alberto Mantovani, and Sebastien Jaillon

Table S1. Cytokine and chemokine levels at the 3-MCA injection site (10 days after 3-MCA administration) and in tumor homogenates, related to Figure 1 and 2.

	**3-MCA injection site** (pg/mg tissue ± SEM)			**Tumor** (ng/mg tissue ± SEM)		
	Csf3r$^{+/+}$	***Csf3r***$^{-/-}$	***p***	***Csf3r***$^{+/+}$	***Csf3r***$^{-/-}$	***p***
**IFNγ**	1.13 ± 0.36	0.272 ± 0.06	**0.0447**	0.11 ± 0.01	0.05 ± 0.01	**0.0075**
**IL-1β**	54.96 ± 12.89	20.57 ± 5.51	**0.0014**	3.83 ± 0.48	1.56 ± 0.29	**0.0007**
**IL-23p19**	49.99 ± 10.56	26.77 ± 10.27	**0.0137**	1.73 ± 0.36	0.75 ± 0.18	**0.028**
**M-CSF**	n.a.	n.a.		0.007 ± 0.001	0.004 ± 0.001	**0.0064**
**IL-22**	17.86 ± 6.14	10.78 ± 4.51	0.6921	0.036 ± 0.001	0.008 ± 0.001	**0.0159**
**IL-12p70**	7.52 ± 2.47	1.13 ± 0.27	**0.0022**	0.18 ± 0.02	0.13 ± 0.01	0.1072
**CXCL1**	89.58 ± 16.14	48.04 ± 8.48	**0.014**	24.03 ± 4.31	66.31 ± 16.52	**0.0291**
**G-CSF**	n.a.	n.a.		0.33 ± 0.08	3.22 ± 0.72	**0.0003**
**CXCL12**	n.a.	n.a.		2.81 ± 0.42	7.07 ± 1.48	**0.0057**
**VEGF**	n.a.	n.a.		0.10 ± 0.01	0.18 ± 0.06	0.2179
**TGFβ**	n.a.	n.a.		0.29 ± 0.06	0.32 ± 0.05	0.6635
**IL-6**	4.03 ± 1.20	9.89 ± 4.12	0.7879	0.25 ± 0.06	0.24 ± 0.06	0.9522
**IL-18**	25.49 ± 2.12	21.78 ± 2.9	0.9588	0.29 ± 0.05	0.30 ± 0.03	0.9588
**CCL2**	36.6 ± 3.80	29.37 ± 7.56	0.6313	23.00 ± 4.62	25.79 ± 3.66	0.6412
**IL-17A**	0.074 ± 0.006	0.994 ± 0.714	0.0931	n.d.	n.d.	
**TNFα**	193.7 ± 31.2	109.7 ± 19.89	**0.0401**	0.54 ± 0.13	1.10 ± 0.10	**0.0043**
**CXCL2**	122.9 ± 24.57	57.33 ± 8.41	**0.014**			

Table S6. Genes used for Type 1 immune signature and for neutrophil-specific signature, related to Figure 7.

Human genes used for Type 1 immune signature	Human genes used for neutrophil signature
IFNG	CSF3R
TBX21	ALPL
STAT1	BST1
STAT4	CD93
IL12RB2	CEACAM3
SOCS1	CPPED1
SOCS2	CREB5
CCR5	CRISPLD2
CXCR3	CXCR1
CD27	CXCR2
IL18RAP	CYP4F3
IRF1	DYSF
IL12A	FCAR
	FCGR3B
	FPR1
	FPR2
	G0S2
	HIST1H2BC
	HPSE
	KCNJ15
	LILRB2
	MGAM
	MME
	PDE4B
	S100A12
	SIGLEC5
	SLC22A4
	SLC25A37
	TECPR2
	TNFRSF10C
	VNN3

Table S7. Demographics and clinicopathologic features of patients from Humanitas Clinical and Research Center with UPS, related to Figure 7.

	N	Median (IQR)
**Age at diagnosis (years)**		
<70	10	61 (66.5-53.25)
≥70	9	73 (78-71)
**Gender**		
Male	14	
Female	5	
**Anatomical site**		
Extremity	8	
Trunk	10	
Retroperitoneal	1	
**Grade**		
G3	19	
